# Phylogeography of a widespread Australian freshwater fish, western carp gudgeon (Eleotridae: *Hypseleotris klunzingeri*): Cryptic species, hybrid zones, and strong intra‐specific divergences

**DOI:** 10.1002/ece3.10682

**Published:** 2023-11-01

**Authors:** Peter J. Unmack, Benjamin D. Cook, Jerald B. Johnson, Michael P. Hammer, Mark Adams

**Affiliations:** ^1^ Centre for Applied Water Science Institute for Applied Ecology, University of Canberra Canberra Australian Capital Territory Australia; ^2^ Australian Rivers Institute, Griffith University Brisbane Queensland Australia; ^3^ frc environmental Wellington Point Queensland Australia; ^4^ Evolutionary Ecology Laboratories, Department of Biology Brigham Young University Provo Utah USA; ^5^ Museum and Art Gallery of the Northern Territory Darwin Northwest Territories Australia; ^6^ Evolutionary Biology Unit South Australian Museum Adelaide South Australia Australia; ^7^ School of Biological Sciences University of Adelaide Adelaide South Australia Australia

**Keywords:** cryptic biodiversity, hybridization, hyper‐cryptic species, introgression, mtDNA, Principal Co‐ordinates Analysis, species delineation

## Abstract

Despite belonging to the most abundant and widespread genus of freshwater fishes in the region, the carp gudgeons of eastern Australia (genus *Hypseleotris*) have proved taxonomically and ecologically problematic to science since the 19th century. Several molecular studies and a recent taxonomic revision have now shed light on the complex biology and evolutionary history that underlies this group. These studies have demonstrated that carp gudgeons include a sexual/unisexual complex (five sexual species plus an assortment of hemiclonal lineages), many members of which also co‐occur with an independent sexual relative, the western carp gudgeon (*H. klunzingeri*). Here, we fill yet another knowledge gap for this important group by presenting a detailed molecular phylogeographic assessment of the western carp gudgeon across its entire and extensive geographic range. We use a suite of nuclear genetic markers (SNPs and allozymes) plus a matrilineal genealogy (cyt*b*) to demonstrate that *H. klunzingeri* s.l. also displays considerable taxonomic and phylogeographic complexity. All molecular datasets concur in recognizing the presence of multiple candidate species, two instances of historic between‐species admixture, and the existence of a natural hybrid zone between two of the three candidate species found in the Murray–Darling Basin. We also discuss the major phylogeographic patterns evident within each taxon. Together, these analyses provide a robust molecular, taxonomic, and distributional framework to underpin future morphological and ecological investigations on this prominent member of regional freshwater ecosystems in eastern Australia.

## Graphical abstract

This study provides a detailed molecular phylogeographic assessment of the western carp gudgeon, a prominent member of freshwater ecosystems across eastern Australia and often found in sympatry with a complex of sexual congeners and their hemiclonal forms. Our comprehensive genetic and genomic data demonstrate the presence of multiple candidate species, instances of historic and contemporary admixture, and pronounced phylogeographic structure within several species.

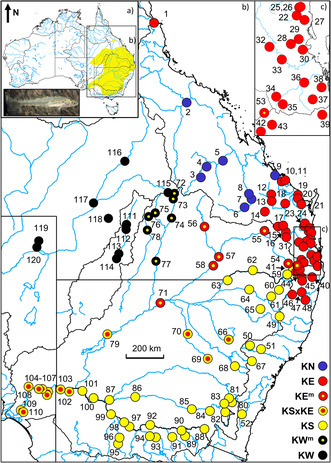

## 1 INTRODUCTION

Phylogeographic assessments of freshwater fishes have commonly revealed cryptic species (e.g., Adams et al., 2014; Baumsteiger et al., 2012; Pinacho‐Pinacho et al., 2018), thereby improving knowledge of species richness and diversity in aquatic ecosystems globally (Seehausen & Wagner, 2014). In Australia, for example, the number of recognized (but not necessarily described) species increased by 39 between 2002 and 2013 (Allen et al., 2002; Unmack, 2013), with many of the newly defined species revealed by molecular phylogenetic and phylogeographic evidence (Adams et al., 2023). Furthermore, phylogeographic studies have indicated that historical, geological, and/or climatic processes can be determinants of contemporary patterns of biodiversity and distribution across riverine landscapes (e.g., Buckley et al., 2021; Shelley et al., 2020; Waters et al., 2020). Consequently, the phylogeographic assessment of freshwater fishes provides foundational diversity and biogeographic knowledge upon which valid ecological and environmental management studies are dependent (Page et al., 2017).

**FIGURE 1:**
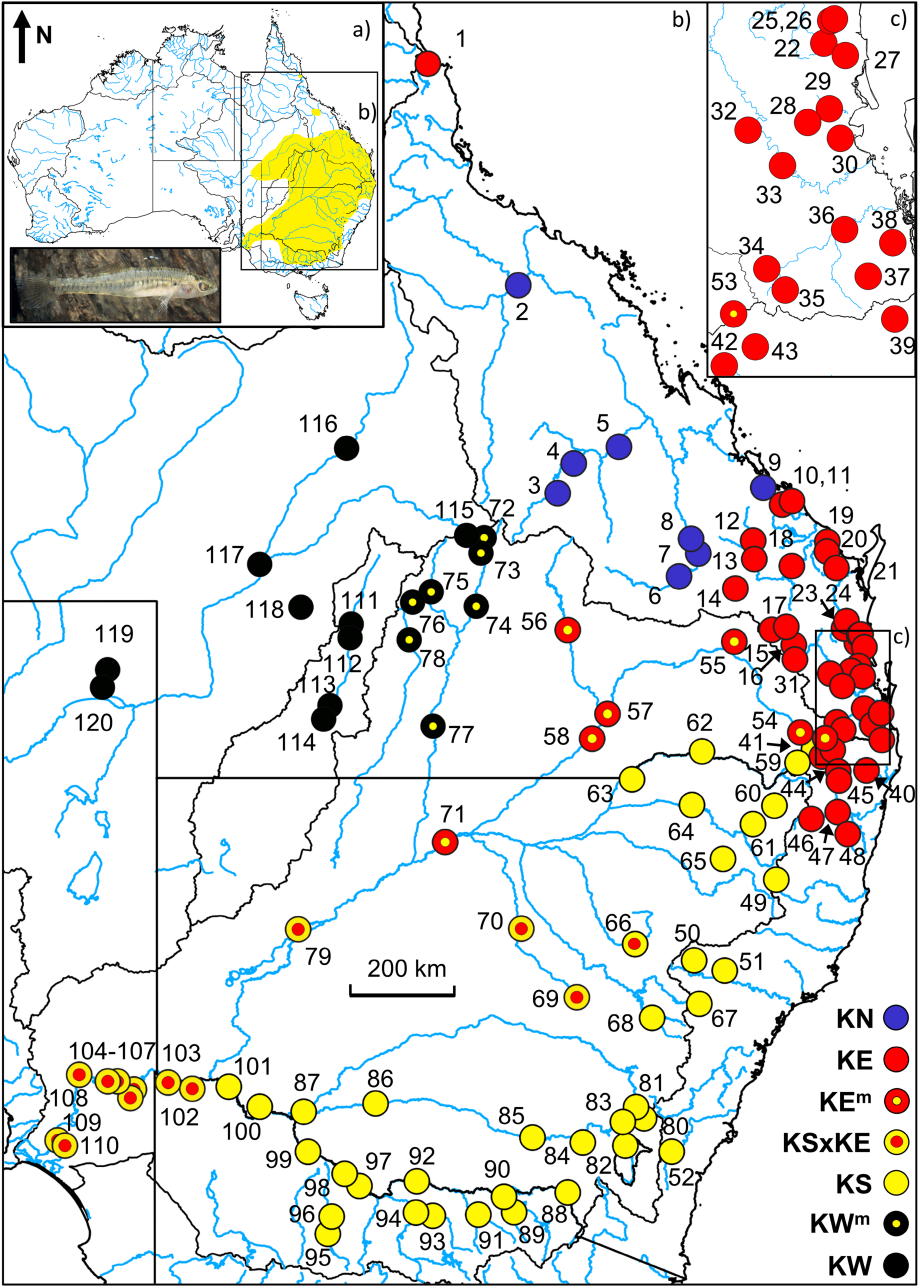
Composite map showing the distribution of *Hypseleotris klunzingeri* and location of all sites surveyed. Each site is represented by a taxon symbol, as per the legend provided and numbered in Table 1. Maps were generated using QGIS v3.8.2 software. Major drainage divisions are outlined with a black border. Photograph shows a male from site 2.

In this study, we present a molecular phylogeographic assessment of the western carp gudgeon (Eleotridae: *Hypseleotris klunzingeri*) throughout its entire Australian range. This small‐bodied freshwater fish (approximate maximum size is 60 mm TL) is one of the most abundant and widely distributed species in eastern Australia (Pusey et al., 2004; Unmack, 2000). Here, it occurs as a disjunct northern population in the Burdekin Basin in central‐northern Queensland, then in coastal drainages from just north of the Fitzroy Basin to the Clarence Basin, as three disjunct southern populations in the Macleay, Hunter, and Shoalhaven basins in central New South Wales, inland throughout the Murray–Darling Basin (MDB), and finally west to the Bulloo River and Cooper Creek in the Lake Eyre Basin (Figure 1, Table 1). *Hypseleotris klunzingeri* is often found in sympatry with other carp gudgeons, all belonging to a species complex comprising a suite of other sexual and hemiclonal congeners, but is reproductively isolated from them (Bertozzi et al., 2000; Schmidt et al., 2011; Unmack et al., 2019).

**TABLE 1:** Site, taxon, and sampling details for all *Hypseleotris klunzingeri* examined in this study.

Site	Taxon	Site code	DD/RB	Region	Locality	State	Latitude	Longitude	SNP^N^	ALZ^N^	CYTB^N^
1	KE	PU1554	I/10	Barron	Groves Ck	Qld	−16.8695	145.5991	2		2
2	KN	PU1535	I/20	Burdekin	Dalrymple Dam	Qld	−20.6315	147.1333	5	5	2
3	KN	PU0152	I/30	Fitzroy	Vandyke Ck	Qld	−24.1636	147.8025	2	3	8
4	KN	PU1436	I/30	Fitzroy	Fairbairn Dam	Qld	−23.6572	148.0749		4	
5	KN	PU0154	I/30	Fitzroy	Mackenzie R	Qld	−23.3750	148.8420	2	2	4
6	KN	PU1202	I/30	Fitzroy	Dawson R	Qld	−25.5744	149.8622	1	2	2
7	KN	PU1203	I/30	Fitzroy	Dawson R	Qld	−25.1869	150.1889	1	2	2
8	KN	Theo	I/30	Fitzroy	Dawson R	Qld	−24.9372	150.0664	2	2	1
9	KN	PU1518	I/33	Boyne	Awoonga Dam	Qld	−24.0670	151.2932	2		2
10	KE	PU0250	I/34	Baffle	Baffle Ck	Qld	−24.3564	151.6119	2	4	4
11	KE	PU1214, PU0242	I/34	Baffle	Oyster Ck	Qld	−24.2963	151.7793	2	1	5
12	KE	PU9958	I/36	Burnett	Three Moon Ck	Qld	−24.9669	151.1208	2	4	7
13	KE	PU0251	I/36	Burnett	Burnett R	Qld	−25.2825	151.1398	2		2
14	KE	PU1446	I/36	Burnett	Auburn R	Qld	−25.7805	150.8188	2	4	2
15	KE	PU1441	I/36	Burnett	Boyne R	Qld	−26.4808	151.4376	2	2	1
16	KE	PU1442	I/36	Burnett	Barkers Ck	Qld	−26.7388	151.8101	2	4	2
17	KE	PU1444	I/36	Burnett	Reedy Ck	Qld	−26.4313	151.6825	2	2	2
18	KE	PU9955	I/36	Burnett	Burnett R	Qld	−25.3989	151.7772	2	5	3
19	KE	PU9751, PU0238	I/37	Burrum	Elliott R	Qld	−24.9872	152.3797	1	2	5
20	KE	PU0237	I/37	Burrum	Gregory R	Qld	−25.1517	152.3719	2	2	4
21	KE	PU0236	I/37	Burrum	Lenthall Dam	Qld	−25.4333	152.5333	2	1	3
22	KE	PU0231	I/38	Mary	Baroon Dam	Qld	−26.7058	152.8803	2	3	5
23	KE	PU9954	I/38	Mary	Yabba Ck	Qld	−26.4592	152.6619	2	7	4
24	KE	PU09114	I/38	Mary	Mary R	Qld	−26.3335	152.7043	2	2	
25	KE	PU09100	I/41	Maroochy	Wappa Dam	Qld	−26.5714	152.9214	2	2	
26	KE	PU09101	I/41	Maroochy	Maroochy R	Qld	−26.5604	152.9438	2		2
27	KE	PU09104	I/41	Maroochy	Ewen Maddock Dam	Qld	−26.7791	153.0089	1	3	
28	KE	PU0996	I/42	Pine	Caboolture R	Qld	−27.0976	152.9137	2	2	
29	KE	PU0987	I/42	Pine	Laceys Ck	Qld	−27.1791	152.7841	2		2
30	KE	PU0988	I/42	Pine	North Pine R	Qld	−27.2736	152.9794	2		1
31	KE	PU9951	I/43	Brisbane	Back Ck	Qld	−26.9875	151.8289	2	8	9
32	KE	PU0990	I/43	Brisbane	Esk Ck	Qld	−27.2255	152.4281	2		1
33	KE	PU0993	I/43	Brisbane	Brisbane R	Qld	−27.4368	152.6336	1	2	2
34	KE	PU9745	I/43	Brisbane	Lake Moogerah	Qld	−28.0533	152.5400	1	6	4
35	KE	PU0228, Lmar	I/45	Albert‐Logan	Lake Maroon	Qld	−28.1800	152.6500	2	6	4
36	KE	PU0979	I/45	Albert‐Logan	Logan R	Qld	−27.8191	153.0044	2	4	2
37	KE	PU0225, PU0224	I/46	South Coast	Coomera R	Qld	−28.0983	153.1456	2	2	2
38	KE	PU0968	I/46	South Coast	Coomera R	Qld	−27.8980	153.2923		3	
39	KE	PU0222	II/1	Tweed	Oxley R	NSW	−28.3539	153.3040	2	1	2
40	KE	PU9942	II/3	Richmond	Richmond R	NSW	−28.8700	153.0442	2	5	2
41	KS	PU14144	II/4	Clarence	Maryvale R	NSW	−28.5093	152.1534	2		1
42	KE	PU14143	II/4	Clarence	Maryvale R	NSW	−28.6335	152.2861	2		2
43	KE	PU14145	II/4	Clarence	Tooloom Ck	NSW	−28.5197	152.4713	2	2	2
44	KE	PU9943	II/4	Clarence	Clarence R	NSW	−28.8875	152.5642	2	3	4
45	KE	PU14148	II/4	Clarence	Clarence R	NSW	−29.0420	152.5766	2		2
46	KE	PU14160	II/4	Clarence	Mann R	NSW	−29.6936	152.1086	2	2	2
47	KE	PU1452	II/4	Clarence	Mann R	NSW	−29.5775	152.5557	2		2
48	KE	PU14153	II/4	Clarence	Nymboida R	NSW	−29.9516	152.7258	2	2	2
49	KS	TR01‐343B, Sali	II/6	Macleay	Salisbury Waters	NSW	−30.7247	151.5131	4	2	4
50	KS	PU1673	II/10	Hunter	Krui R	NSW	−32.0964	150.1183	2		2
51	KS	PU1417	II/10	Hunter	Wybong Ck	NSW	−32.2692	150.6381	2	2	4
52	KS	PU1345A	II/15	Shoalhaven	Shoalhaven R	NSW	−35.3427	149.7381	5		5
53	KE^m^	PU1412	IV/22	MDB:Condamine	Condamine R	Qld	−28.3231	152.3418	2		2
54	KE^m^	PU1414	IV/22	MDB:Condamine	Leslie Dam	Qld	−28.2248	151.9212		1	
55	KE^m^	PU1440	IV/22	MDB:Condamine	Charleys Ck	Qld	−26.6840	150.8007	2	2	2
56	KE^m^	PU9960, PU1438	IV/22	MDB:Condamine	Maranoa R	Qld	−26.4869	147.9733	2	7	6
57	KE^m^	PU14138	IV/22	MDB:Condamine	Balonne R	Qld	−27.9105	148.6488		3	
58	KE^m^	PU14139	IV/22	MDB:Condamine	Balonne R	Qld	−28.3303	148.3873	2	2	
59	KS	PU9949, Seve	IV/16	MDB:Border	Severn R	Qld	−28.7400	151.8736	2	4	5
60	KS	PU1459	IV/16	MDB:Border	Severn R	NSW	−29.4690	151.4820	2		2
61	KS	PU1332	IV/16	MDB:Border	McIntyre R	NSW	−29.7812	151.1173	2		2
62	KS	PU1334	IV/16	MDB:Border	McIntyre R	NSW	−28.5472	150.2527	2	2	2
63	KS	PU1637	IV/16	MDB:Border	Boomi R	NSW	−29.0217	149.0641	1		1
64	KS	PU1328	IV/18	MDB:Gwydir	Gwydir R	NSW	−29.4570	150.0821	2		2
65	KS	PU1330	IV/19	MDB:Namoi	Manilla R	NSW	−30.3723	150.6087	1		1
66	KSxKE	PU1336	IV/20	MDB:Castlereagh	Castlereagh R	NSW	−31.8186	149.1172	1		1
67	KS	PU9970	IV/21	MDB:Macquarie	Dunns Swamp	NSW	−32.8344	150.2064	2	6	4
68	KS	PU0254	IV/21	MDB:Macquarie	Turon R	NSW	−33.0725	149.4061	1	1	4
69	KSxKE	PU14122	IV/21	MDB:Bogan	Bogan R	NSW	−32.7226	148.1275	1		1
70	KSxKE	Bog	IV/21	MDB:Bogan	Bogan R	NSW	−31.5572	147.1852		2	
71	KE^m^	PU1325, PU1421	IV/25	MDB:Darling	Darling R	NSW	−30.0870	145.8930	1	1	2
72	KW^m^	PU1431	IV/23	MDB:Warrego	Nive R	Qld	−24.9182	146.5549	2	2	2
73	KW^m^	Nive	IV/23	MDB:Warrego	Nive R	QLD	−25.1800	146.5000		5	
74	KW^m^	PU1503	IV/23	MDB:Warrego	Warrego R	QLD	−26.0820	146.4203	2	3	
75	KW^m^	Walk	IV/23	MDB:Warrego	Walker Ck	QLD	−25.8380	145.6550		1	
76	KW^m^	PU97105	IV/23	MDB:Warrego	Ambathala Ck	Qld	−26.0130	145.3400	2	7	4
77	KW^m^	PU9963	IV/23	MDB:Warrego	Warrego R	Qld	−28.1181	145.6867	2	6	4
78	KW^m^	PU9961	IV/24	MDB:Paroo	Paroo R	Qld	−26.6514	145.2819	2	3	4
79	KSxKE	PU1317	IV/25	MDB:Darling	Darling R	NSW	−31.5680	143.3978	1	1	1
80	KS	PU1303	IV/12	MDB:Lachlan	Meadow Ck	NSW	−34.7789	149.2686	2	2	2
81	KS	PU1304, PU1305	IV/12	MDB:Lachlan	Blakney Ck	NSW	−34.5861	149.1333		2	
82	KS	PU1482	IV/10	MDB:Murrumbidgee	Murrumbidgee R	ACT	−35.2422	148.9517	5		5
83	KS	PU1302	IV/10	MDB:Murrumbidgee	Yass R	NSW	−34.8385	148.9086	2	2	2
84	KS	PU1309	IV/10	MDB:Murrumbidgee	Killimicat Ck	NSW	−35.1882	148.2286	2	2	2
85	KS	PU1341A	IV/10	MDB:Murrumbidgee	Murrumbidgee R	NSW	−35.1048	147.3759	2		2
86	KS	PU1338A	IV/10	MDB:Murrumbidgee	Murrumbidgee R	NSW	−34.5259	144.7119	1		1
87	KS	PU1340A	IV/10	MDB:Murrumbidgee	Murrumbidgee R	NSW	−34.6656	143.4914	1		1
88	KS	PU1338B	IV/1	MDB:Upper Murray	Murray R	NSW	−36.0375	147.9729	2		2
89	KS	PU1592	IV/2	MDB:Kiewa	Bight Ck	Vic	−36.3733	147.0628	2	2	
90	KS	PU0821	IV/9	MDB:Mid Murray	Wodonga Ck	Vic	−36.1099	146.8914		3	
91	KS	PU1360	IV/3	MDB:Ovens	Ovens R	Vic	−36.4132	146.4556	2		2
92	KS	PU0822	IV/9	MDB:Mid Murray	Ulupna Ck	Vic	−35.8481	145.4116	2		2
93	KS	PU1361	IV/4	MDB:Broken	Broken R	Vic	−36.4330	145.6840	2	2	2
94	KS	PU1362	IV/5	MDB:Goulburn	Goulburn R	Vic	−36.3783	145.3969	2		2
95	KS	PU1479	IV/7	MDB:Lodden	Loddon R	Vic	−36.7409	143.9011	2		2
96	KS	PU1385, PU17115	IV/7	MDB:Lodden	Loddon R	Vic	−36.4458	143.9666	2		2, 5*
97	KS	PU9437	IV/9	MDB:Mid Murray	Murray R	Vic	−35.9261	144.4375	1	3	3
98	KS	PU9436	IV/9	MDB:Mid Murray	Black Swamp	Vic	−35.7194	144.1875		2	7
99	KS	PU0824	IV/9	MDB:Mid Murray	Little Murray R	Vic	−35.3442	143.5647	2	2	
100	KS	PU17106	IV/9	MDB:Mid Murray	Murray R	Vic	−34.5803	142.7451			3*
101	KS	PU0825	IV/14	MDB:Lower Murray	Kings Billabong	Vic	−34.2409	142.2200	2		2
102	KSxKE	PU0826	IV/14	MDB:Lower Murray	Lake Cullulleraine	Vic	−34.2758	141.6007	2		2
103	KSxKE	Lind	IV/14	MDB:Lower Murray	Murray R	Vic	−34.1700	141.1900	2	3	1
104	KSxKE	PU17107	IV/26	MDB:Lower Murray	Murray R	SA	−34.2874	140.6105			14*
105	KSxKE	Yabb	IV/26	MDB:Lower Murray	Murray R	SA	−34.4333	140.5500	1		1
106	KSxKE	Over	IV/26	MDB:Lower Murray	Murray R	SA	−34.1500	140.3300	3		2
107	KSxKE	Devl	IV/26	MDB:Lower Murray	Murray R	SA	−34.1500	140.1667	3		1
108	KSxKE	Morg	IV/26	MDB:Lower Murray	Murray R	SA	−34.0377	139.6813		1	
109	KSxKE	PU0830	IV/26	MDB:Lower Murray	Murray R	SA	−35.1529	139.3150	2	2	1
110	KSxKE	PU17114	IV/26	MDB:Lower Murray	Murray R	SA	−35.2414	139.4390			4*
111	KW	PU1426	XI/1	Bulloo	Bulloo R	Qld	−26.3842	144.2985	2	2	2
112	KW	PU9962	XI/1	Bulloo	Bulloo R	Qld	−26.6178	144.2783	2	7	9
113	KW	PU1425	XI/1	Bulloo	Bulloo R	Qld	−27.7676	143.9372	2		2
114	KW	Bull	XI/1	Bulloo	Bulloo R	Qld	−28.0000	143.8300	1	1	1
115	KW	PU97103	X/3	Cooper	Barcoo R	Qld	−24.8786	146.2567	1	8	6
116	KW	Darr	X/3	Cooper	Darr R	Qld	−23.4000	144.2200	1	1	2
117	KW	PU1427	X/3	Cooper	Cooper Ck	Qld	−25.3700	142.7446	2	1	2
118	KW	PU1428	X/3	Cooper	Kyabra Ck	Qld	−26.0974	143.4445	2	2	
119	KW	Coon	X/3	Cooper	Brown Ck	SA	−27.1609	140.1638	1	3	3
120	KW	Nari	X/3	Cooper	Cooper Ck	SA	−27.4589	140.0758	2	1	2
									204	233	293

*Note*: ^N^Sample sizes are shown for each molecular dataset (ALZ, allozymes).

Abbreviations: DD, Australian Drainage Division; RB, River Basin.

* Half cyt*b* sequences only.

As reviewed by Pusey et al. (2004), *H. klunzingeri* occurs in a wide range of lotic and lentic habitats, with aquatic plants and leaf‐litter beds, and slow‐flowing water (i.e., <0.2 m/s) the preferred (but not exclusively used) habitat features. The species is primarily benthic, with a diet dominated by microcrustaceans and macroinvertebrates (e.g., chironomids, ephemeropterans, and trichotperans). *Hypseleotris klunzingeri* has a wide range of water quality tolerances but is only known from upper estuarine areas downstream of tidal barrages that prevent upstream movement, indicating that estuarine habitats are not naturally used by the species. Spawning can occur most of the year (excluding winter), but peaks in spring and early summer in response to increasing water temperature and daylight length, and possibly flow events, with flow events reported to catalyze the onset of mass migration of *H. klunzingeri* (Pusey et al., 2004).

As with many other Australian freshwater fishes, the taxonomy and distribution of *H. klunzingeri* has historically been equivocal, having first been mistaken by Klunzinger (1872, 1880) as *H. cyprinoides* (which does not occur in Australia), then described as *Carrassiops klunzingeri* by Ogilby (1898), to be later reassigned to the genus *Hypseleotris*. Its common name of western carp gudgeon relates to the early thought that the taxon primarily occurred west of the Great Dividing Range compared with the more easterly *H. galii* (e.g., Anderson et al., 1971), whereas today we know that representatives of both lineages naturally occur both east and west of the Great Dividing Range (Thacker, Geiger, & Unmack, 2022). While the current nomenclature suggests a robust taxonomic status, matrilineal phylogeographic data presented by Thacker et al. (2007) indicated several divergent lineages within *H. klunzingeri* that to date have not been closely re‐assessed. Furthermore, their phylogeographic analyses suggest the low elevation divide between the coastal Burnett River and the Condamine River in the northern MDB is the pathway by which *H. klunzingeri* was exchanged between these basins (see also Unmack, 2013).

The purpose of this study is to present a thorough molecular phylogeographic assessment of *H. klunzingeri*, using comprehensive sampling and an expanded suite of molecular datasets. Regarding the latter, our choice of two independent sets of co‐dominant nuclear markers (single nucleotide polymorphisms [SNPs] and allozyme loci), plus matrilineal sequence data (the mtDNA gene Cytochrome *b* [cyt*b*]), ensures that this study is ideally placed to help resolve the above‐described taxonomic uncertainty and re‐evaluate biogeographic patterns in this prominent Australian freshwater fish.

## 2 MATERIALS AND METHODS

### 2.1 Sample collection

All sampling was approved by the University of Canberra Committee for Ethics in Animal Experimentation (approval codes CEAE 13‐06, 15‐06 and 20180442) and undertaken under the following state wildlife collecting licenses: New South Wales P07/0007‐5.0 and P18/0027‐1.1; Queensland 168221, 191126 and 212524; South Australia S115 and ME9902959; Victoria RP1146 and RP1344. Fish were ethically euthanized using either AQUI‐STM or dilute clove oil, snap‐frozen in liquid nitrogen, and deposited in the SA Museum's Australian Biological Tissues Collection.

We sampled throughout range of *H. klunzingeri* for a total of 120 sites (Figure 1, Table 1). All river names used herein are cross‐referenced to site numbers in Table 1. Each molecular dataset has a different number of populations and individuals but overlap between marker types was high. The SNP dataset consisted of 106 sites and 204 individuals, allozymes had 80 sites and 233 individuals, and cyt*b* included 101 sites and 293 individuals (Table 1). The cyt*b* dataset includes all *H. klunzingeri* sequences from Thacker et al. (2007). Two versions of the cyt*b* dataset were constructed, one for the whole gene with 267 individuals, and a second one for the first 601 base pairs to accommodate an additional 26 individuals from four locations (marked in Table 1 with an asterisk) that provided additional phylogeographic insights within the MDB using these larger sample sizes. For ease of presentation, individuals are identified throughout by their final taxon assignment, namely, pure taxa KN, KE, KS, and KW (reflecting their various compass orientations), plus assorted hybrid/admixed combinations thereof (KE^m^ = KE from the MDB, KW^m^ = KW from the MDB, and KSxKE = hybrids between KS and KE).

### 2.2 SNP genotyping

DNA was extracted by Diversity Arrays Technologies (DArT Pty Ltd, Canberra, Australia, www.diversityarrays.com) using a NucleoMag 96 Tissue Kit (Macherey‐Nagel) coupled with NucleoMag SEP to allow automated separation of high‐quality DNA on a Freedom Evo robotic liquid handler (TERAN Pty Ltd).

Sequencing for SNP genotyping was done using DArTseq™ (DArT Pty Ltd), which uses a combination of complexity reduction using restriction enzymes, implicit fragment size selection, and next‐generation sequencing (Sansaloni et al., 2011), as described in detail by Kilian et al. (2012). The technique is similar to double‐digest restriction associated DNA sequencing (ddRAD) (Peterson et al., 2012), but has the advantages of accepting lower quantities of DNA, greater tolerance of lower quality DNA, and higher call rates (Sansaloni et al., 2011). The restriction enzyme combination of PstI (recognition sequence 5′‐CTGCA|G‐3′) and SphI (5′‐GCATG|C‐3′) was used for the double digestion.

The PstI‐compatible adapter included the Illumina flowcell attachment sequence, a sequencing primer sequence, a barcode region of variable length (see Elshire et al., 2011), and the PstI‐compatible overhang sequence. The reverse adapter contained flowcell attachment sequence and SphI‐compatible overhang sequence. Only fragments generated by the PstI‐SphI double digest were effectively amplified by polymerase chain reaction (PCR) (Georges et al., 2018).

Sequences generated from each lane were processed using proprietary DArT analytical pipelines as outlined by Georges et al. (2018) to yield repeatable SNP markers. In addition, DArT processes approximately one‐third of the samples twice from DNA to allelic calls as technical replicates and scoring consistency (repeatability) was used as the main selection criterion for high quality/low error rate markers.

### 2.3 SNP filtering

After receiving the SNP data from DArT Pty Ltd, the SNP data and associated metadata were read into a genlight object as implemented in R package adegenet (Jombart, 2008) to facilitate subsequent processing with R package dartR (Gruber et al., 2018). We created two different datasets based upon different filtering of the initial 19,903 polymorphic SNP loci, one for the phylogenetic analysis (‘phylo’ dataset), and the other for the PCoA and fixed difference analyses (‘PCoA’ dataset). The phylo dataset was initially filtered to remove any obviously introgressed individuals within the MDB (identified using the PCoA dataset), as reticulation events are not compatible with bifurcating trees. The next step retained only loci for which repeatability was greater than 0.99 and all loci with a callrate above 0.6. The PCoA dataset included all individuals and was first filtered for repeatability to include values >0.99. The second filtering step removed all secondary loci (loci found within the same sequenced fragment) with the locus retained having the higher polymorphism information content (PIC) value. Finally, loci with a callrate above 0.9 were retained. The additional filtering steps were undertaken on the PCoA dataset for the two analyses that are sensitive to the presence of too many missing values and/or tightly linked loci (ordination and the calculation of fixed differences). The data remaining after these primary filtering steps for both datasets are regarded as highly reliable. The PCoA dataset was used for each of the additional (stepwise) PCoA analyses based on a subset of individuals being compared, with additional filtering applied to remove any loci that become monomorphic in such subsets.

### 2.4 SNP analyses

Genetic similarity among individuals and populations was visualized using ordination (Principal Coordinates Analysis [PCoA]; Gower, 1966), using individuals as entities and loci as attributes and implemented by the gl.pcoa and gl.pcoa.plot functions of dartR. For our phylogenetic analysis, we constructed SNP genotypes for each individual by concatenating only the variable bases from each SNP locus into a single partition. A few loci had the SNP removed with the adaptor, because of chance matching of the adaptor sequence to the terminal region containing the SNP. These loci were removed prior to concatenation. Heterozygous SNP positions were represented by the standard ambiguity codes. We generated a phylogenetic tree using maximum likelihood (ML) applied to concatenated sequences. ML analyses were conducted using RAxML 8.2.12 (Stamatakis, 2014) on the CIPRES cluster (Miller et al., 2010) using the model GTRCAT and searching for the best‐scoring ML tree using the model GTRGAMMA in a single program run, with bootstrapping set to finish based on the autoMRE majority rule criterion. The tree was imported to Mega 7.0.18 (Kumar et al., 2016), formatted, and mid‐point rooted. To assist with identifying potential introgressed individuals, heterozygosity was calculated in R using the command “het <‐ rowMeans(as.matrix(gl)==1, na.rm=T)” followed by “write.csv (het, file=“het.csv”).”

The diagnosability of lineages and candidate species was assessed by calculating the number of pairwise fixed differences (both absolute and allowing a 5% tolerance for shared alleles at each locus) and the associated probabilities that such values could arise through sampling error alone (dartR command gl.fixed.diff; parameter tloc = 0 or tloc = 0.05; see Unmack et al., 2022 for rationale and methods involved).

### 2.5 Allozyme genotyping and analyses

Our allozyme dataset comprised the same 54 putative allozyme loci as employed by Unmack et al. (2019) and was generated according to the principles and procedures presented in Richardson et al. (1986) and Hammer et al. (2007). We used PCoA, coupled with assessments of diagnosability (fixed differences, allowing a 10% cumulative tolerance for shared alleles at a locus as advocated by Adams et al., 2014, for allozyme markers) and admixture (intermediate positioning between parental taxa for PCoA, higher levels of heterozygosity, and lack of fixed differences at otherwise diagnostic loci), to explore the broader taxonomic and phylogeographic patterns evident in this dataset. The rationale and methods for these analyses follow Adams et al. (2014) and Unmack et al. (2022).

### 2.6 MtDNA genotyping and analyses

The mitochondrial cyt*b* gene was sequenced following the PCR protocols in Hammer et al. (2014), except that samples were amplified with the following primer pairs: Glu18 TAACCAGGACTAATGRCTTGAA with Hd.alt GGRTTGTTGGAGCCTGTTTCAT or Hd.Hyps GGGTTGTTGGAGCCSGTTTCGT and midg.496 GGCGGCTTTTCRGTAGATAA with Eleo.Thr.40 GATTTTAACCTCCTGCGTCCG. Sequences were edited using Chromas 2.6.5 (Technelysium) and imported into BioEdit 7.2.5 (Hall, 1999). Sequences were aligned by eye and checked via amino acid coding in MEGA to test for unexpected frame shift errors or stop codons. Data were analyzed phylogenetically using RAxML as per for the SNP analysis but using the model GTRGAMMA.

## 3 RESULTS

### 3.1 Identification of primary taxa and lineages

#### 3.1.1 Ordination of SNPs

**FIGURE 2:**
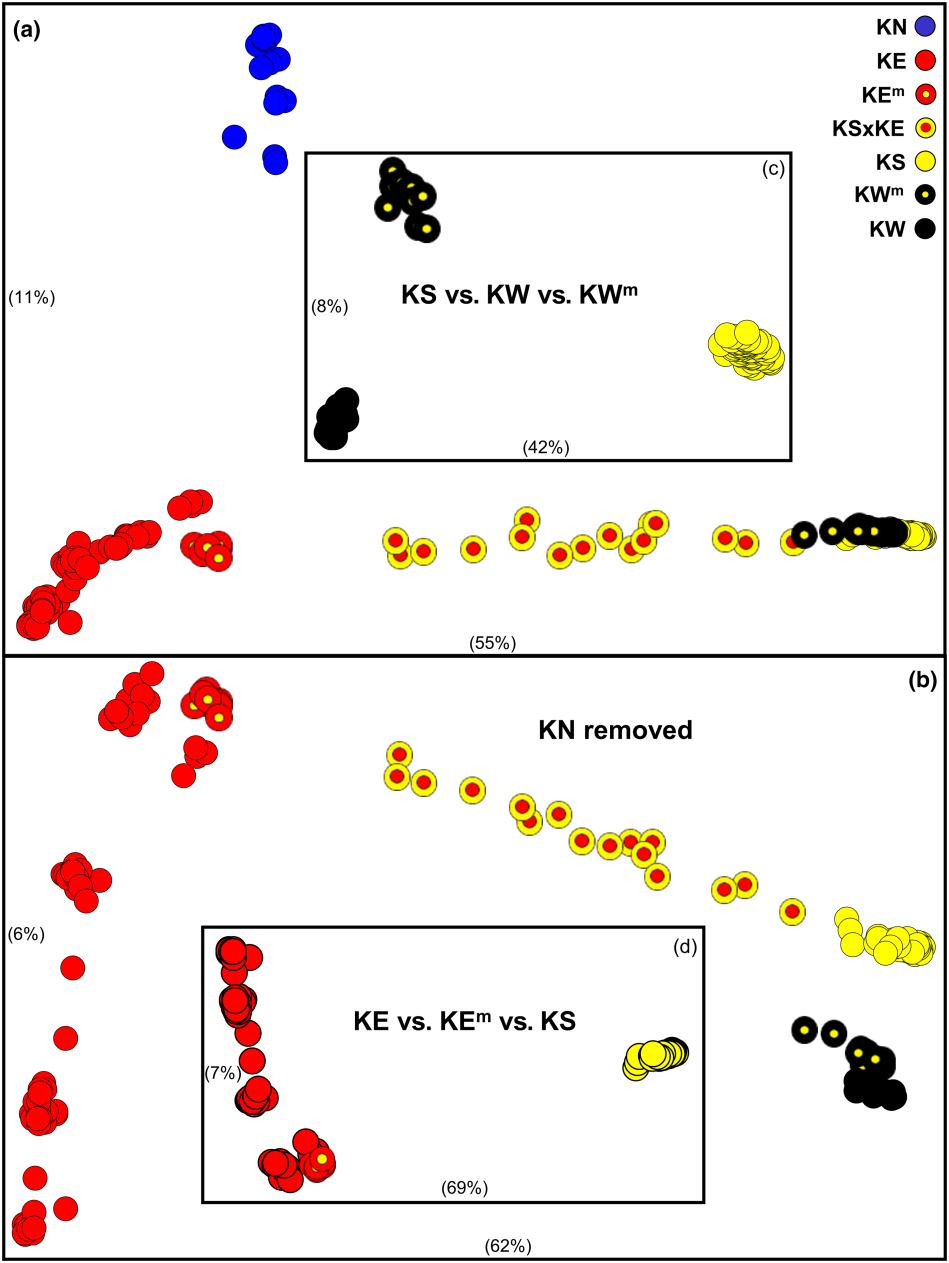
Scatterplots of ordination scores in the first two dimensions for the initial PCoA and three follow‐up PCoAs for the stringently filtered SNP dataset. The relative contribution of each dimension is given in brackets (axes not scaled accordingly). (a) Initial PCoA of all 204 individuals (3628 SNPs, 3.4% missing data); (b) PCoA after the removal of KN (*n* = 189, 3342 SNPs, 2.4% missing data); (c) PCoA of the KS, KW, and KW^m^ individuals (*n* = 92, 1511 SNPs, 1.8% missing data); (d) PCoA for KE, KE^m^, and KS (*n* = 105, 2511 SNPs, 1.7% missing data). Symbols as for Figure 1.

An initial PCoA of the stringently filtered ‘PCoA’ dataset revealed three primary clusters, ultimately referable to the taxon groupings KN, KE/KE^m^, and KS/KW/KW^m^, plus a series of 16 admixed individuals, which linked the latter two clusters in the first dimension (Figure 2a). Further targeted PCoAs (Figure 2b; Figure S1), supported by all other molecular datasets, consistently demonstrated that these admixed individuals, representing populations from the Bogan and Castlereagh Rivers (Darling River tributaries), the lower Darling River, and Murray River populations downstream from the Darling River junction, mark the presence of at least one hybrid zone between pure KS and pure KE. These additional PCoAs also revealed that pure KS individuals were readily diagnosable from those occurring in the northwestern inland rivers (Figure 1) and further revealed that this latter taxon itself comprised two allied but distinctive lineages, pure KW in the Cooper and Bulloo systems, and KW^m^ in two MDB rivers, the Warrego and Paroo (Figure 2b,c). Although only subtly supported by PCoA (Figure 2; Figure S1), it became evident across all other analyses that the KE individuals in the upper MDB (Condamine system down to the mid‐Darling River) displayed modest levels of introgression with KS. In consequence, we also assigned a unique taxon identifier (KE^m^) to individuals from these sites.

Strong support for the presence of four primary taxa (KN, KE, KS, and KW) plus three KS‐admixed groupings in the MDB (KE^m^, KSxKE, and KW^m^) is also evident in the fixed difference counts (Table 2). These data clearly demonstrate that the primary taxa are all readily diagnosable at multiple loci (range 38–432 absolute fixed differences; 64–525 near‐fixed differences), whereas KSxKE displays no fixed differences from either parental taxon (as expected for recently admixed populations) and both KE^m^ and KW^m^ display fewer fixed differences from KS than do their pure parental taxa (as typically found for historic admixture/introgression). Additional support is presented by the observed heterozygosity counts for each group (Table 2), which show all pure taxa display comparatively low levels of heterozygosity (range 0.0098–0.0218) when compared to KE^m^ (almost double that of pure KE), KW^m^ (more than double that of pure KW), and most notably KSxKE (more than fourfold higher than either parent).

**TABLE 2:** Pairwise number of diagnostic SNP loci between the primary genetic groups identified by PCoA (Figure 2).

Taxon	KE (72)	KE^m^ (9)	KN (15)	KS (66)	KSxKE (16)	KW^m^ (10)	KW (16)
KE	–	3628	3553	3628	3628	3623	3618
KE^m^	**0** ^**ns**^/1	–	3553	3628	3628	3623	3618
KN	106/194	155/193	–	3553	3553	3548	3543
KS	93/344	60/182	224/455	–	3628	3623	3618
KSxKE	**0** ^**ns**^ **/2** ^**ns**^	**0** ^**ns**^ **/1** ^**ns**^	110/156	**0** ^**ns**^ **/0** ^**ns**^	–	3623	3618
KW^m^	141/310	115/181	291/429	**12** ^**ns**^/40	**7** ^**ns**^/25	–	3613
KW	260/414	225/247	432/525	38/64	20/39	14/23	–
*H*_O_	0.0218	0.0387	0.0175	0.0161	0.0919	0.0228	0.0098

*Note*: Lower triangle = number of absolute fixed differences/number of fixed differences allowing 5% tolerance for shared alleles (tloc = 0.05); upper triangle = number of SNPs for each pairwise comparison. All values were highly significant (*p* < .001) after Bonferroni correction for multiple tests except where indicated (bold font and superscript^ns^). Also shown are the observed heterozygosity counts for each group.

#### 3.1.2 Phylogenetic analysis of SNPs

**FIGURE 3:**
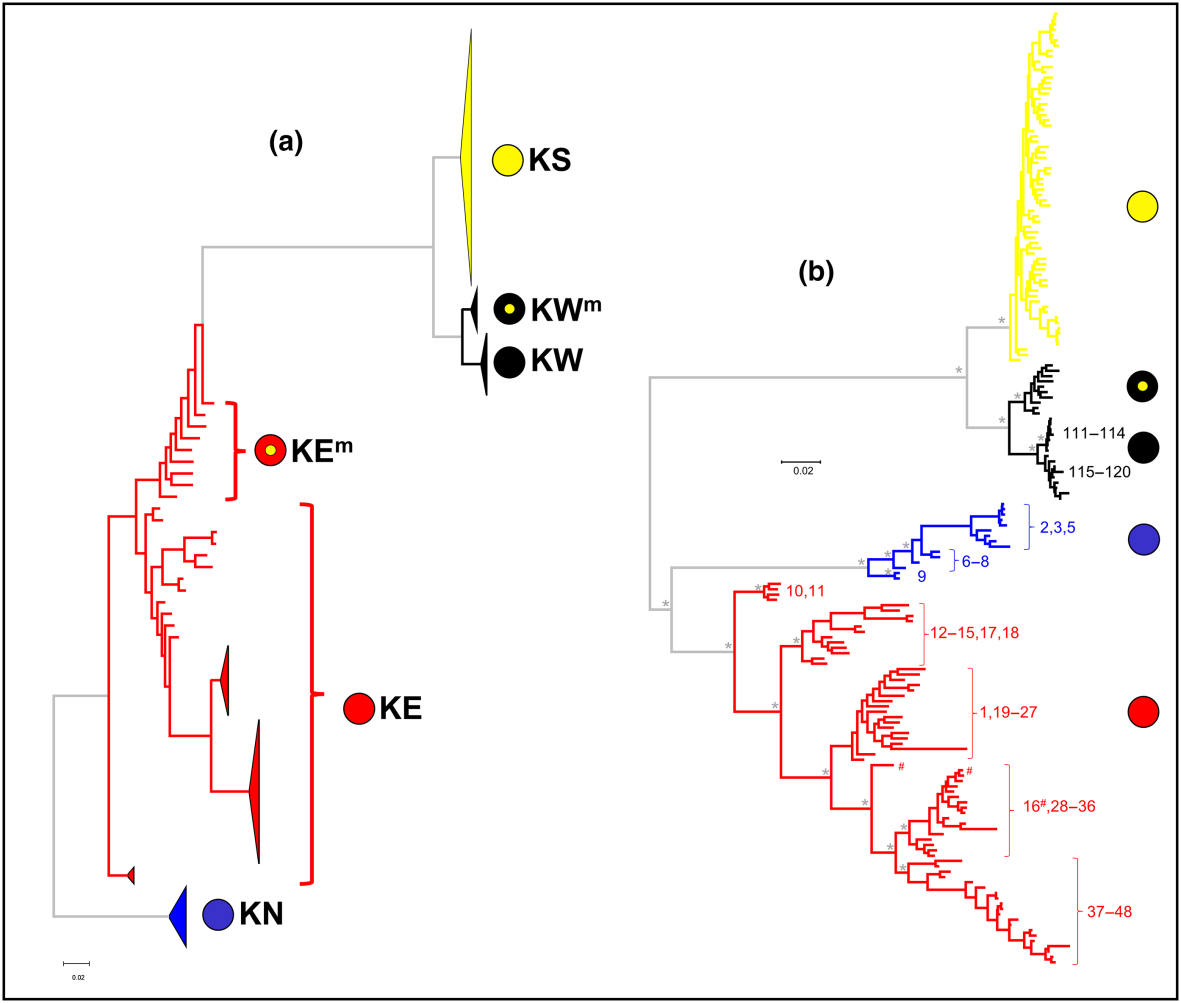
RAxML trees for SNP dataset. (a) Preliminary tree skeleton, showing how the inclusion of KE^m^ individuals distorts relationships within KE and blurs the distinctiveness of KE and KS. (b) Final RAxML tree, rooted at the mid‐point. Branches are color coded by primary taxon and major clades identified by the symbols used following Figure 1. Early‐branching nodes with bootstrap values of 95% or higher are asterisked. Minor clades within taxa are labeled with their corresponding site codes. (# = site 16, the only Burnett fish not aligning with the other Burnett sites).

Our initial ‘phylo’ dataset comprised all individuals except for the 16 hybrid KSxKE fish. Surprisingly, the presence of the nine admixed KE^m^ individuals caused considerable distortion of the relationships among populations and taxa in the resultant ML tree (Figure 3a), reflecting the reality that reticulate evolution often leads to data that do not exhibit tree‐like behavior (Unmack et al., 2022). Removal of these KE^m^ individuals followed by re‐filtering resulted in a final ‘phylo’ dataset of 179 individuals for 7419 polymorphic SNP loci.

ML recovered one tree with a −ln score of −72522.995738 and the rapid bootstrap search finished at 450 replicates (Figure 3b; full tree in Figure S2). Support across most of the deeper nodes of the tree was strong, with once again four principal lineages recognized, namely, KN and KE (as sister clades), with both being sister to clades KS and KW. Taxon KN represents northern coastal populations from the Burdekin, Fitzroy, and the coastal Boyne rivers (note there are two Boyne rivers in our study, the second being a tributary to the Burnett River). Apart from its presence in the Condamine and Darling (as lineage KE^m^), taxon KE consists of populations from eastern coastal rivers from Baffle Creek south to the Clarence River, plus a recently introduced population in the Barron River in far north Queensland (site 1). Taxon KS contains individuals from three coastal New South Wales rivers (Macleay, Hunter, and Shoalhaven) along with non‐introgressed populations from the MDB, except those in the Warrego and Paroo rivers (lineage KW^m^), which, along with Bulloo River and Cooper Creek populations, are referable to taxon KW. Outside of the MDB, most individuals tend to group closely with others from the same or adjacent river and there is obvious phylogeographic structure at the regional level in all taxa except KS.

#### 3.1.3 Allozyme analyses

**FIGURE 4:**
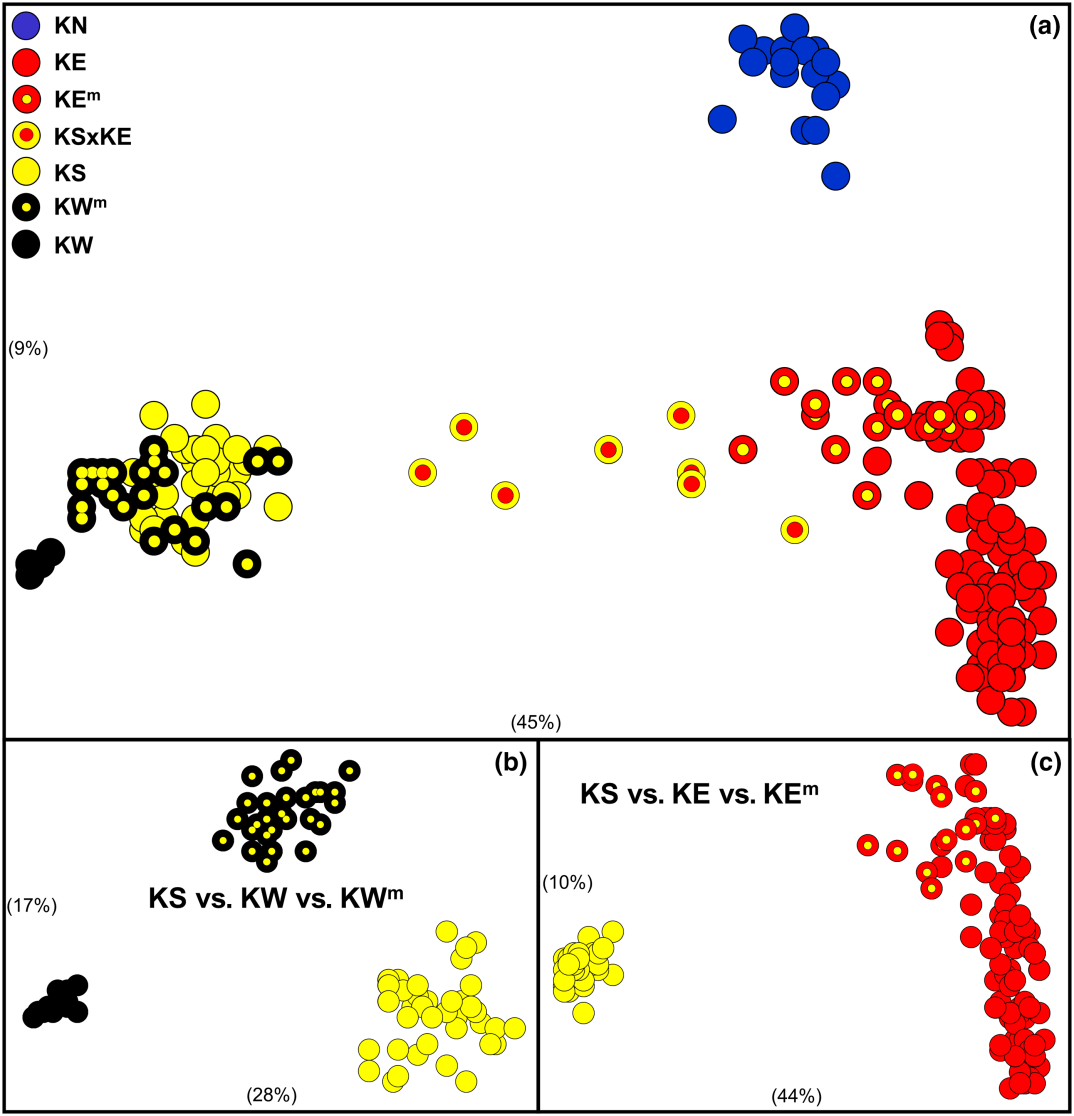
Scatterplots of ordination scores in the first two dimensions for the initial PCoA and two follow‐up PCoAs for the allozyme dataset. (a) initial PCoA of all 233 individuals; (b) PCoA of the 92 individuals referable to KS, KW, or KW^m^; (c) PCoA of the 151 individuals referable to KE, KE^m^, or KS. Symbols and presentation as for Figure 2.

**FIGURE 5:**
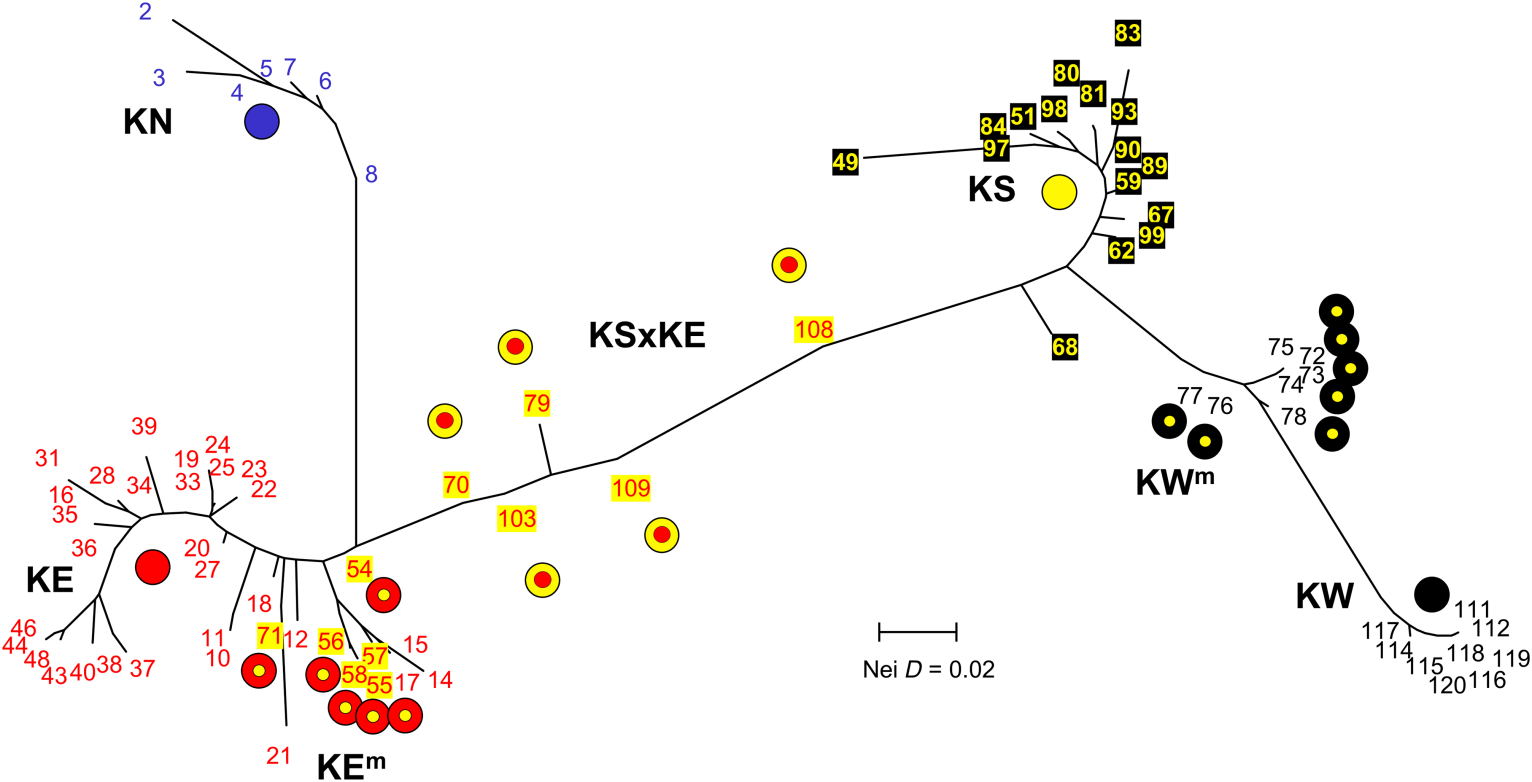
Neighbor‐joining tree based on pairwise Nei Distances among all sites surveyed in the allozyme study.

The results of our allozyme analyses closely mirrored those obtained for the SNPs dataset, both in terms of primary genetic lineages and identifying pure versus admixed populations. A series of PCoA analyses of the allozyme data (Figure 4) displayed a near‐identical association between individuals as depicted for the SNPs (Figure 2), and together supported the presence of the same seven primary groupings, namely, KN, pure KE, KE^m^ (close to pure KE but slightly displaced toward KS), KSxKE, pure KS, KW^m^, and pure KW. The same assignment of sites into primary groupings is shown in an unrooted NJ tree (Figure 5). These findings are further validated by the fixed difference and observed heterozygosity counts for each grouping (Table 3), which show the same patterns of diagnosability and comparative levels of heterozygosity as found for the SNPs. Together, our two nuclear datasets fully support the presence of four primary taxa in the western carp gudgeon, namely, KN, KE+ (KE + KE^m^), KS, and KW+ (KW + KW^m^), all readily diagnosable by numerous SNP and allozyme loci (Table 4).

**TABLE 3:** Pairwise number of diagnostic allozyme loci between the primary genetic groups identified by PCoA.

Lineage	KN (20)	KE (96)	KE^m^ (16)	KSxKE (9)	KS (39)	KW^m^ (27)	KW (26)
KN	–	0.16	0.14	0.15	0.29	0.31	0.41
KE	4	–	0.04	0.07	0.28	0.30	0.41
KE^m^	4	0	–	0.04	0.23	0.25	0.38
KSxKE	5	0	0	–	0.08	0.10	0.18
KS	12	11	5	0	–	0.05	0.09
KW^m^	12	9	5	1	1	–	0.07
KW	18	17	16	4	3	2	–
*H*_O_	0.060	0.059	0.134	0.182	0.090	0.074	0.017
±SE	0.016	0.010	0.026	0.033	0.021	0.018	0.008

*Note*: Lower = number of fixed differences (10% tolerance for all shared alleles combined); upper = unbiased Nei's distance. Also shown are the observed heterozygosity counts (*H*_O_) and standard errors (SE) for each group.

**TABLE 4:** Summary of outcomes from applying the framework recommended by Unmack et al. (2022) to assess which lineages of *H. klunzingeri* sensu lato are also candidate species.

Pairwise comparison	Diagnostic molecular markers			Comparative distribution	Sampling intensity	Candidate
	SNP	Alloz	Cyt*b*			Species?
KN vs. KE+	93/186	4	+++	Parapatry/widespread/no barrier	Adequate vs. strong	Yes
KN vs. KS	224/455	12	+++	Allopatry/moderate/genuine gap	Adequate vs. strong	Yes
KN vs. KW+	264/443	14	+++	Allopatry/shallow	Adequate vs. strong	Yes
KE+ vs. KS	39/313	10	+	Parapatry/partial	Strong vs. strong	Yes
KE+ vs. KW+	64/296	11	+++	Allopatry/shallow	Strong vs. strong	Yes
KS vs. KW+	6*/37	1	++	Allopatry/moderate/genuine gap	Strong vs. strong	Probably

*Note*: Diagnostic molecular markers: SNPs = number of absolute fixed differences/number of fixed differences (5% tolerance); all values but one are highly statistically significant (*p* < .001; *p = .026): Alloz = number of fixed differences (10% tolerance): Cyt*b* = +++ unequivocally diagnosable, numerous fixed nucleotide differences; ++ = unequivocally diagnosable, some fixed nucleotide differences; + distinct primary clades but not unequivocally diagnostic. Terminology for comparative geographic distribution follows Figure S5 (see also Unmack et al., 2022). Sampling intensity: the extent to which each lineage has been geographically sampled (all pairwise comparisons reflect intense genomic sampling).

#### 3.1.4 MtDNA analyses

**FIGURE 6:**
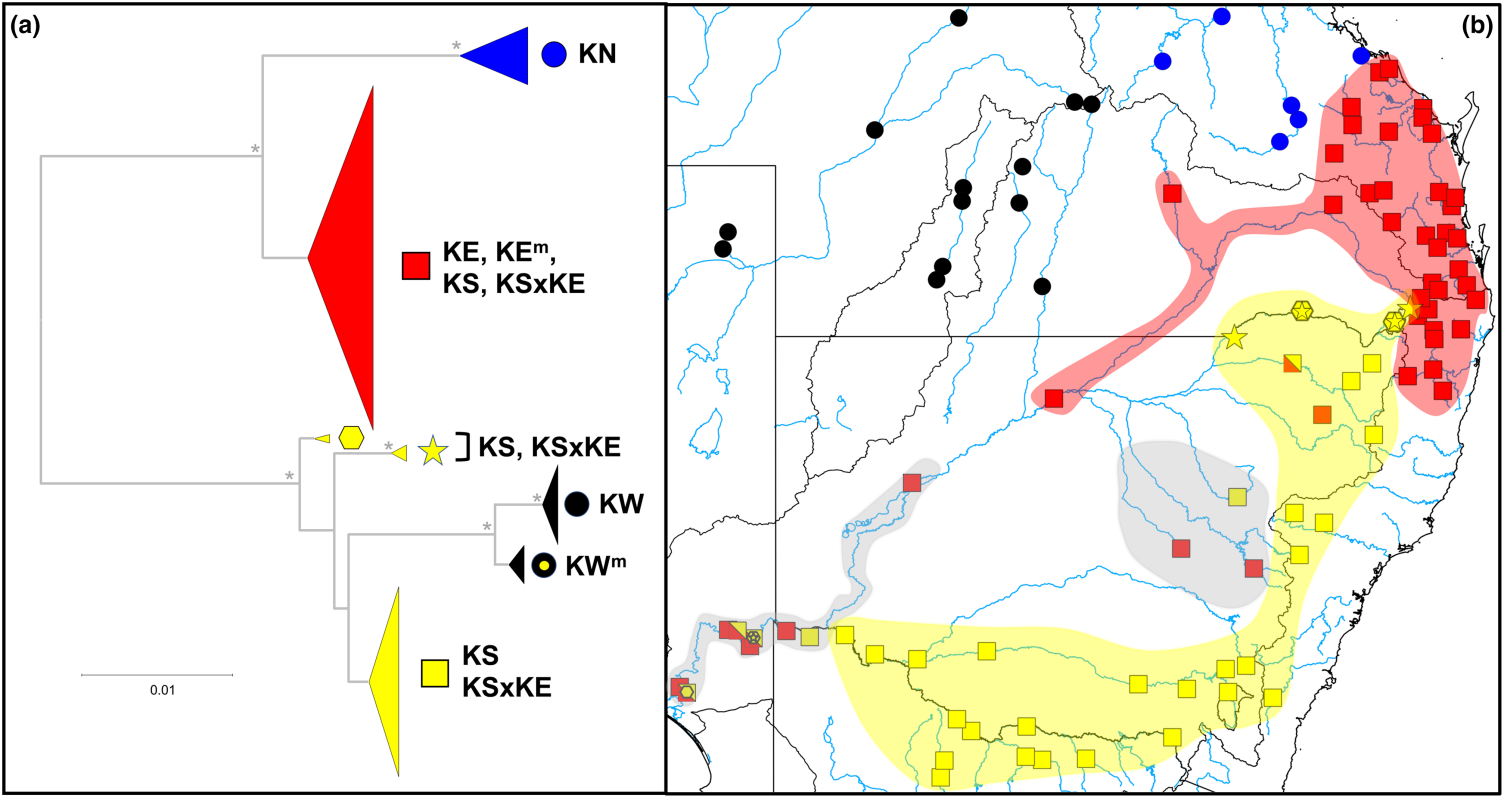
Summary of mtDNA analyses for *Hypseleotris klunzingeri*. (a) Condensed gene tree for full cyt*b* sequences. Early‐branching nodes with bootstrap values of 95% or higher are asterisked. MtDNA clade symbols match those used in (b). Detailed tree presented in Figure S3. (b) Map of the major cyt*b* lineages in the MDB and adjacent drainages. Shading represents the geographic distribution identified in this study for candidate species KS (yellow) and KE (red), and for the two KSxKE hybrid zones (gray).

The whole and half cyt*b* datasets consisted of 1141 and 601 base pairs for 267 and 293 individuals, respectively. Maximum likelihood (ML) was run on the two cyt*b* datasets with RAxML producing trees with likelihood scores of −3145.296216 and −1595.307571, and the rapid bootstrap search for both analyses finishing at 650 and 500 replicates, respectively (simplified tree for whole cyt*b* in Figure 6; detailed trees for both whole and half cyt*b* in Figures S3 and S4, respectively).

For the full cyt*b* dataset, the deeper phylogenetic relationships were moderately well‐supported and largely similar to those found for SNPs. The KN clade was sister to the KE clade and both were sister to a composite KS/KW clade, comprising three distinctive sublineages referable to taxon KS plus well‐supported, sister sublineages for KW and KW^m^ (Figure 6a; Figure S3). Consistent with their hybrid status, KSxKE individuals displayed either KS‐derived or KE‐derived haplotypes (ratio 14:15, Figure S4). For the non‐hybrid taxa, an individual's mtDNA clade membership was concordant with their SNP/allozyme primary lineage identification in all instances apart from six KS fish, all from three northern MDB rivers (sites 65, 66, and 68; Gwydir, Namoi, and Macquarie Rivers).

Mapping the distribution of the major cyt*b* lineages (Figure 6) clearly demonstrates that KE‐derived haplotypes have intruded into the KSxKE hybrid zones as identified using our nuclear datasets (Figure 1 and shaded in Figure 6) plus are present in the pure KS populations from some upper MDB rivers. It also reveals that haplotypes from two rare KS‐lineages, otherwise characteristic of the Border Rivers in the upper MDB (sites 59, 62, and 63), have also spread across the drainage divide into the upper Clarence River (site 41) and far downstream into the KSxKE hybrid zone in the lower Murray.

### 3.2 Origin and dynamics of KSxKE hybrid zones

Our molecular data provide several additional perspectives on the KSxKE hybrid zones. Consistent with geographic expectations, stepwise PCoA of the SNPs dataset consistently identified the Condamine (i.e., taxon KE^m^) as the most likely source population for the KE+ parent (Figure 3b; Figure S1). Second, individuals displayed varying degrees of admixture between their two parental taxa (Figure S1), indicating that F_1_ hybrids must be sufficiently fertile to at least produce F_2_ and/or backcross offspring. Finally, there was no obvious correlation between the distance from the KE^m^ source and the extent of introgression for KE‐derived alleles (Figure 1; Figure S1).

### 3.3 Within‐taxon phylogeographic structure

**FIGURE 7:**
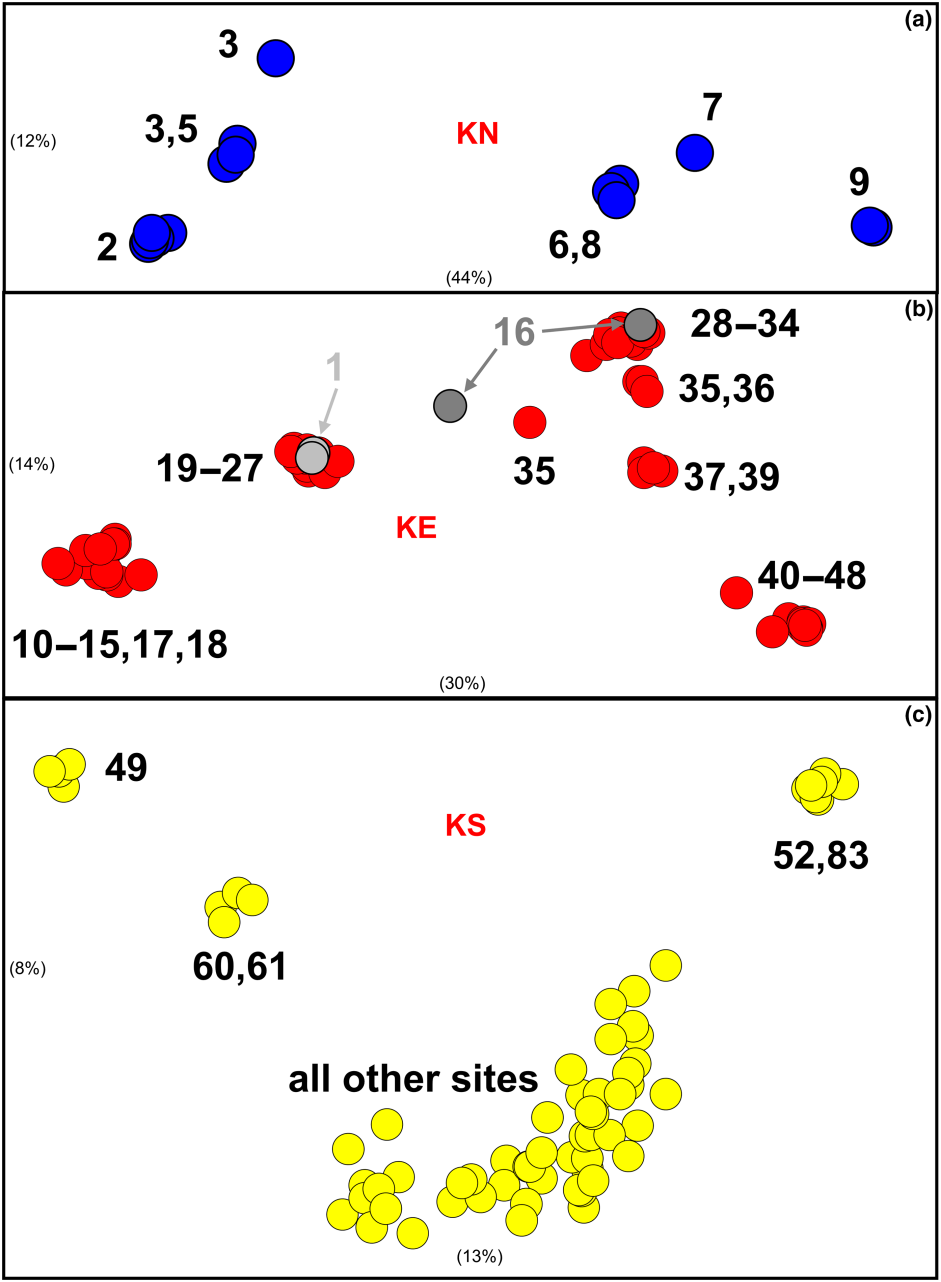
Results of PCoA for the refiltered SNP datasets for pure populations of taxa KN, KE, and KS. Axes are scaled according to the relative contribution of each dimension (in brackets). Clusters are labeled with site codes. (a) KN (*n* = 15); (b) KE (not including KE^m^ individuals; *n* = 72); (c) KS (*n* = 66).

Both our SNP and mtDNA datasets contain sufficient genetic insights to explore phylogeographic trends within the four primary taxa. In addition to the well‐supported dichotomy between KW and KW^m^, the SNPs identified a shallow split in KW between Bulloo and Cooper populations (sites 111–114 vs. 115–120; Figure 3), They also revealed additional albeit more complicated phylogeographic structure for the other three taxa (Figures 3 and 4), herein further explored using taxon‐specific PCoAs (Figure 7).

For KN, there was a primary dichotomy between the three northern and four southern sites, which reflects latitude rather than river basin membership (Figure 7a). Most notably, the Burdekin KN was closely allied with those in the Fitzroy River (Figures 3 and 7) despite being somewhat of a northern outlier (Figure 1).

A more complex pattern is evident within KE+. All relevant PCoAs (Figures 2b,d and 4c; Figure S1) consistently found that Condamine fish (KE^m^) were genetically most similar to those in the Burnett, while two geographic outliers (sites 1 and 16) and a number of regional population clusters were present for pure KE in the RAxML tree (Figure 3b). A KE‐specific PCoA revealed a similar pattern of diversity, including the site 1 and 16 outliers (discussed separately), but was also able to detect a primary phylogeographic split between sites from the Maroochy River northward and west to the Burnett (sites 10–29, excluding site 16) versus sites south of and including the Pine and Brisbane Rivers (sites 28–48). The northern outlier (site 1, Barron River) clearly clusters with sites 19–24 (Burrum and Mary Rivers), supporting its likely status as an introduced population from that part of KE's range. Intriguingly, both individuals from site 16 (Burnett River) are anomalously placed, one intermediate between the two primary clusters (and showing elevated heterozygosity levels) and the other clustering with the southern phylogroup. This same pattern is displayed in the allozyme data (PCoA not shown) and in the mtDNA tree (Figure S3), with one individual from site 16 clustering with Brisbane River haplotypes (southern phylogroup) and the other with Burnett River haplotypes (northern phylogroup).

With respect to KS, most sites are relatively homogeneous, with only modest structure relating to geographic outlying populations in two of the Border Rivers (Severn and McIntyre Rivers; sites 60, 61), the Macleay River (site 49), and the Shoalhaven River (site 52), the latter clustering with one of the sites in the adjacent drainage divide (site 83, Murrumbidgee River) and therefore inferring a source population. Although the mtDNA data are also relatively homogeneous for KS, the Border Rivers harbored two distinctive cyt*b* lineages at high frequency that were absent elsewhere in pure KS (Figure 6).

## 4 DISCUSSION

Building on the work of Thacker et al. (2007), we present three comprehensive molecular datasets that together identify four primary taxa (KN, KE+, KS, and KW+) plus several examples of historic (KE^m^, KW^m^) and relatively recent admixture (KSxKE) within the parent species *H. klunzingeri*. All primary taxa, lineages, and admixed zones are fully diagnosable by numerous independent genetic markers, and our intensive sampling provides an ideal starting point for future field surveys to plug apparent distributional gaps, ecological assessments of the *H. klunzingeri* complex, or formal taxonomic revision. Regarding the latter, allocating the nominal form of *H. klunzingeri* s.s. (Ogilby, 1898) to a specific taxon may prove problematic, given the type locality (the Murray River in South Australia) is apparently, at least the time of collecting for genetic evaluation, a hybrid zone (KSxKE).

### 4.1 Candidate species

The genetic resolution of candidate species has often relied on identifying lineages using either gene genealogies (e.g., mtDNA or nDNA gene trees) or multilocus population trees (for allozymes or genomic data). However, as such tree‐only approaches detect genetic structure rather than candidate species per se (Sukumaran & Knowles, 2017; Unmack et al., 2022), lineages delineated in this manner need not directly equate to biological or evolutionary species but may instead reflect major phylogeographic breaks within a species or a composite of two or more species plus admixed individuals.

While there is no simple formula for deciding whether two genetically distinctive allopatric populations are conspecific or represent different species, we have recently advocated a six‐step approach to assist in this task (Unmack et al., 2022). These steps are as follows: identify lineages, hybrids, and introgressed populations using a combination of ordination of individuals (step 1) plus phylogenetic methods (step 2), followed by pairwise assessments of lineage diagnosability (step 3), comparative geographic distribution (step 4), and sampling intensity (step 5), and concluding with a review of any other biological information that might indicate that lineages are not conspecific (step 6). Unfortunately, observations relevant to this final step are largely unavailable in the literature, since many ecological studies of *Hypseleotris* in eastern Australia have not attempted to reliably distinguish *H. klunzingeri* from a suite of congeneric and often co‐occurring taxa (e.g., Lintermans, 2007; Meredith et al., 2003), now known to comprise a complex of sexual species and ‘unisexual’ (hybridogenetic) lineages (Thacker et al., 2022b; Unmack et al., 2019). We hope that a recent taxonomic revision by Thacker, Geiger, and Unmack (2022) for this hemiclonal species complex, which includes five sexual species and multiple unisexual combinations, will help establish a more robust taxonomic framework for identifying individuals to their correct sexual group and hence facilitate the documentation of comparative biological information for all sexual forms of *Hypseleotris*, including those referable to the *H. klunzingeri* complex.

Table 4 summarizes the outcomes of applying steps 3–5 to the primary taxa identified for *H. klunzingeri* using steps 1 and 2. As shown, there is strong evidence that KN, KE+, and KS are all valid candidate species, being unequivocally or effectively diagnosable from each other at hundreds of unlinked genes and displaying distributional patterns that are inconsistent with being phylogeographic lineages within a single species (Table 4; Figure S5). Given their comparatively low number of diagnostic differences, the decision as to whether the allopatric taxa KS and KW+ are conspecific or represent distinct evolutionary species remains the only taxonomic question not fully resolved by our stand‐alone genetic datasets. However, as the number of molecular characters that diagnose KS from KW+ greatly exceeds the nine partially diagnostic morphological characters that delineate other co‐occurring species of *Hypseleotris* (Thacker, Geiger, & Unmack, 2022), we have concluded that KW+ ‘probably’ represents a fourth candidate species. A full resolution of its taxonomic status will require additional targeted assessments of any morphological and other biological differences between KS and KW+, and must include exemplars of pure KS, pure KW, and KW^m^. The scenario of sister Cooper versus MDB candidate taxa is also evident in another co‐occurring freshwater fish (Australian smelt, *Retropinna* spp.; Unmack et al., 2022).

### 4.2 Broad patterns within and between candidate species

Taxon KN has an unusual distribution and genetic pattern. The southern portion of the Fitzroy River Basin is quite different to the northern portion, a pattern not replicated in any species examined so far. The species is unknown from coastal basins between the Fitzroy and Burdekin Basins except for the small Herbert Creek catchment (tissues were not available for this study). This absence appears to be real, with moderately intensive sampling only finding its congener *H. bucephala* at many sites. Within the Burdekin Basin, KN has a narrow distribution, being found primarily in Lake Dalrymple and in the Burdekin River upstream of the dam until the vicinity of Charters Towers. It is absent from the arid Belyando River, the main southern tributary to Lake Dalrymple and containing habitats that are otherwise commonly inhabited by *H. klunzingeri*. Again, *H. bucephala* is present at many sites across the entire Burdekin Basin. Either KN was once widespread in coastal basins north to the Burdekin Basin, or it crossed over the drainage divide between the Fitzroy and Burdekin Basins, such as via the Rugby portal (Georges et al., 2018) with subsequent extirpation of intervening populations. It is also not fully possible to rule out that KN has been translocated to the Burdekin Basin from the northern Fitzroy Basin as fish stocking contaminants.

The separation between taxa KN and KE corresponds to the Boyne River and Baffle Creek catchment boundary. This separation is also found between two rainbowfish species (*Melanotaenia splendida* and *M. duboulayi*), as well as two hardyhead species (*Craterocephalus fulvus* and *C. stercusmuscarum*), plus is represented by a major disjunction in *Hypseleotris acropinna*. It also represents the northern most distribution points of two other species (*Retropinna semoni* and *Philypnodon macrostomus*), which do not occur north of Baffle Creek (Unmack, 2001). Taxon KE is continuously distributed and common in most streams from Baffle Creek south to the Clarence River. Populations are primarily structured by river basin, with a deeper divergence between Baffle Creek, Burnett River, the Burrum system south to the Maroochy River, and populations from the Caboolture River south to the Clarence River. Two populations represented geographic outliers. The first is a clearly introduced population in the Barron Basin (site 1), with a likely origin from the Burrum Basin. The second is the presence of fish in Barkers Creek (site 16, Burnett system) that display both nuclear and matrilinear evidence of admixture between two otherwise distinctive ‘northern’ and ‘southern’ phylogroups. It is unclear from our data if this represents a natural occurrence or a past translocation event.

Complex geographic and genetic patterns are found for *H. klunzingeri* populations within the MDB. Befitting its extensive geographic coverage and low‐relief topography, the basin harbors the pure taxa KS, KE+, and KW+, the latter two showing evidence of historic admixture with KS (as lineages KE^m^ and KW^m^), plus a relatively recent and possibly ongoing hybrid zone (KSxKE) between upstream KE+ and its downstream congener KS. Within KS, there is no strong pattern of phylogeographic structure. These populations are primarily found in the Murray subcatchment upstream of the Darling River confluence, along with the Macquarie, Gwydir, Namoi, and Macintyre subcatchments. Taxon KE+ is restricted to the Condamine–Balonne subcatchment. These KE^m^ lineage individuals have obvious genetic affinities to populations from the Burnett River, a common pattern for those MDB species that are also found in coastal river basins in southeastern Queensland (Unmack, 2013). Fish with the KSxKE genetic profile are present in the Darling River south into the lower Murray River, plus in the Castlereagh and Bogan subcatchments. These are likely a result of KE^m^ fish from the Balonne River dispersing further downstream, but also managing to push upstream into nearby tributaries like the Bogan and Castlereagh. Contemporary patterns in the Darling River are likely to vary over time as drought eliminates populations (due to excess water extraction), with recolonization either coming via floodwaters from either the Balonne (introducing more KE^m^ fish) or Macintyre/Barwon rivers (taxon KS fish), carrying different genotypes into the lower Darling River. The western portion of the Murray–Darling Basin in the Warrego and Paroo subcatchments has admixed populations between KS and KW, represented by KW^m^. These populations share a long drainage basin boundary with both Bulloo River and Cooper Creek (which contains pure KW). One other fish species has crossed from the Lake Eyre Basin rivers into the Paroo and Warrego subcatchments, *Melanotaenia splendida* (Lintermans, 2007), while the turtle *Emydura macquarii* has crossed from the Bulloo River into Paroo (Georges et al., 2018). The most likely spot for faunal exchange is via the Bindegolly portal (Georges et al., 2018).

There have been four invasions into east coast river basins of KS from the MDB. There first is located in the upper Maryvale River in the upper Clarence Basin (site 41), which has a mitochondrial haplotype identical to those adjacent in the Border Rivers subcatchment (upper Macintyre River), along with a similar relationship based on SNPs (Figures S2 and S3). The second invasion occurred in the upper Macleay River in Salisbury Waters (site 49), which is adjacent to the Gwydir subcatchment. The fish from Salisbury Waters are most similar to those from the Border Rivers subcatchment in the upper Macintyre River for SNPs, while for mitochondrial DNA from the upper Macintyre and the Gwydir, subcatchments were similar. The third transfer occurred with the Hunter Basin. This population has long been considered likely native as they are known to be widespread, although patchy in occurrence, and several other fishes are shared with the Hunter, but not in surrounding coastal basins (e.g., *Craterocephalus amniculus*, *Mogurnda adspersa*), or they are also present in some additional surrounding basins (e.g., *Tandanus tandanus*). Hunter Basin KS (sites 50, 51) had a clear genetic affinity with fish from the Murrumbidgee subcatchment rather than the adjacent Macquarie and Namoi subcatchments. The fourth coastal basin population was found in the Shoalhaven Basin (site 52). This population was genetically closest to fish primarily from the adjacent Murrumbidgee subcatchment.

It is tempting to speculate on whether these four geographic outliers represent native populations or human‐mediated introductions. While many such introductions likely remain undocumented, over 50 Australian freshwater fishes are already known or presumed to have been either deliberately or accidentally introduced into catchments outside their native range (Lintermans, 2004). If these four coastal KS populations were native, we would expect each to show the greatest genetic similarity to its adjacent MDB population. This is the case for three of these comparisons, with the Hunter Basin being the exception. In addition, we might expect native occurrences to have broader distributions within coastal basins provided they have had a large period of time to disperse. Instead, all but the Hunter appear to only harbor localized populations, which have not dispersed far. Introductions could come from nearby populations, thus mimicking a native pattern, or they could be from distant populations, if accidentally introduced along with deliberately stocked, hatchery‐reared sportfish. Unfortunately, each of these basins lacks early historical records, a common situation for Australia's freshwater fishes. At this stage, we consider these four populations are likely introduced, although we acknowledge the evidence is equivocal.

The presence of three out of the four candidate species in the MDB is an unusual distributional pattern. The majority of species of native fish known to occur in the MDB do not share the Basin with widely distributed and truly sibling congeners, the exceptions being *Craterocephalus fluviatilis* (Murray Hardyhead) and *C. amniculus* (Darling Hardyhead) and several *Galaxias* species in the mountain galaxiid complex, which are closely related (Lintermans, 2007; Raadik, 2014). Some introgression has been recorded in both fish groups (Adams et al., 2011, 2014). There are also three congeneric species groups present in the MDB that are known to produce hybrids from the genera *Maccullochella* (Douglas et al., 1995) and *Philypnodon* (Hammer et al., 2019). In other groups, there are examples of introgression such as in the genus *Melanotaenia* between three species (P. J. Unmack, M. Adams, unpublished data), along with an admixture zone in the genus *Retropinna* (Hammer et al., 2007; Unmack et al., 2022). In addition, there is the hemiclonal complex of *Hypseleotris* carp gudgeons, which have hybrid origins (Unmack et al., 2019). Given that even distantly related fish species are known to readily hybridize (Vespoor & Hammar, 1991), the MDB provides considerable opportunities for mixing gene pools from different colonizations and reinvasions of the basin from surrounding river basins over evolutionary time frames across a range of species with different levels of genetic divergence. It is also likely that opportunities for hybridization have increased as natural habitats in the MDB have been anthropogenically altered or degraded (Lintermans, 2007; Scribner et al., 2001).

### 4.3 Cryptic biodiversity in Australian freshwater fishes

The resolution of cryptic species diversity within *H. klunzingeri* accords with the trend of finding new candidate species in Australian freshwater fish guided by molecular data (e.g., Adams et al., 2014; Hammer et al., 2019; Unmack et al., 2022). Such data can not only reveal the presence of cryptic species but can also uncover nuanced evidence for admixture and introgression that was often not detectable prior to the advent of detailed genomic datasets and the coupling of ordination and tree‐based approaches. The application of this modern molecular approach to taxonomic uncertainty in *H. klunzingeri* has identified the presence of additional candidate species in this nominal taxon. Moreover, as in our companion study of Australian smelt (*Retropinna* spp.; Unmack et al., 2022), additional morphological, phenotypic, or ecological data are not required in most cases to validate the identified candidate species per se (the exceptions being KW+ vs. KS in *Hypseleotris*, and COO vs. MTV in *Retropinna*). However, such data remain the foundation for formal description and naming of candidate species and are of course valuable for addressing other questions about the biology of individual species, whether formally named or not.

## 5 CONCLUSIONS

This study has revealed that carp gudgeons are even more speciose than previously thought, adding several additional candidates to the existing six sexual species plus their various hemiclonal relatives (Thacker, Geiger, & Unmack, 2022, Thacker, Shelley, et al., 2022; Unmack et al., 2019). Even ignoring the complication of sympatric hemiclones, many river basins contain at least three or more sexual species, with the geographically extensive MDB notably harboring six sexual taxa (plus multiple hemiclones). Moreover, the observed partial mismatch between geographic and phylogenetic patterns, plus the presence of a natural hybrid zone in the lower Murray and in at least two Darling tributaries add yet more layers of complication. Given such complexity, future taxonomic and field identification efforts will be particularly challenging and ideally require the involvement of a molecular identification technology (with SNPs providing the gold standard of unequivocal identification all sexual and unisexual forms) as part of a coordinated accumulation of companion morphological exemplars of each morphotype at each site surveyed. Our own research group has already adopted this strategy where resources permit.

The dynamic boom and bust nature of many Australian freshwater ecosystems highlights the need for monitoring spatial genetic patterns for all resident species over time, particularly after major climate events such as have impacted eastern Australia over the past decade (Hughes et al., 2009; Legge et al., 2022; Lintermans, 2013). Our study provides future researchers with a framework to pursue such an endeavor for the *Hypseleotris* of eastern Australia. As a major component of the biodiversity and ecology of these ecosystems, carp gudgeons also offer great potential for environmental monitoring, provided researchers can identify individuals to their correct taxon. In the past, carp gudgeons have often been lumped into one composite ‘taxon’ (*Hypseleotris* spp.) when included in ecological surveys (Lintermans, 2007), a custom that precludes any genuine assessment of whether *Hypseleotris* alpha diversity has declined or shifted (e.g., hemiclone ratio/presence) at such sites. Finally, this study further underlines the point that active conservation and management practices for freshwater fishes, including both the intended (i.e., wrong genetic lineage used) and unintended (i.e., where carp gudgeons or other non‐target species unknowingly contaminate the hatchery release event) consequences of fish stocking programs (Lintermans, 2004), need to be mindful of the existence of both undescribed candidate species, and deep phylogeographic structure within all species, to avoid undertaking or facilitating translocations or mixing of distinct genetic lineages.

## AUTHOR CONTRIBUTIONS

**Peter J. Unmack:** Conceptualization (equal); formal analysis (equal); funding acquisition (equal); investigation (equal); methodology (equal); project administration (equal); resources (equal); writing – original draft (equal); writing – review and editing (equal). **Benjamin D. Cook:** Conceptualization (supporting); writing – original draft (equal); writing – review and editing (supporting). **Jerald B. Johnson:** Funding acquisition (supporting); project administration (supporting); resources (supporting); writing – review and editing (supporting). **Michael P. Hammer:** Conceptualization (supporting); investigation (supporting); resources (supporting); writing – review and editing (equal). **Mark Adams:** Conceptualization (equal); data curation (equal); formal analysis (equal); funding acquisition (equal); investigation (equal); methodology (equal); resources (equal); writing – original draft (equal); writing – review and editing (equal).

## Supporting information


Figure S1

Figure S2

Figure S3

Figure S4

Figure S5

Data S1

## Data Availability

The allozyme, genomic, and mtDNA sequence data are available on Zenodo (https://doi.org/10.5281/zenodo.8223411). All novel cyt*b* sequences have also been deposited in GenBank (accession numbers OR401349–OR401588).
