# Supplementary figures and images for: Phylogeography of a widespread Australian freshwater fish, western carp gudgeon (Eleotridae: Hypseleotris klunzingeri): Cryptic species, hybrid zones, and strong intra‐specific divergences
Source: Ecol Evol. 2023 Nov 1;13(11):e10682. doi: 10.1002/ece3.10682 (PMC10618717; doi:10.1002/ece3.10682)

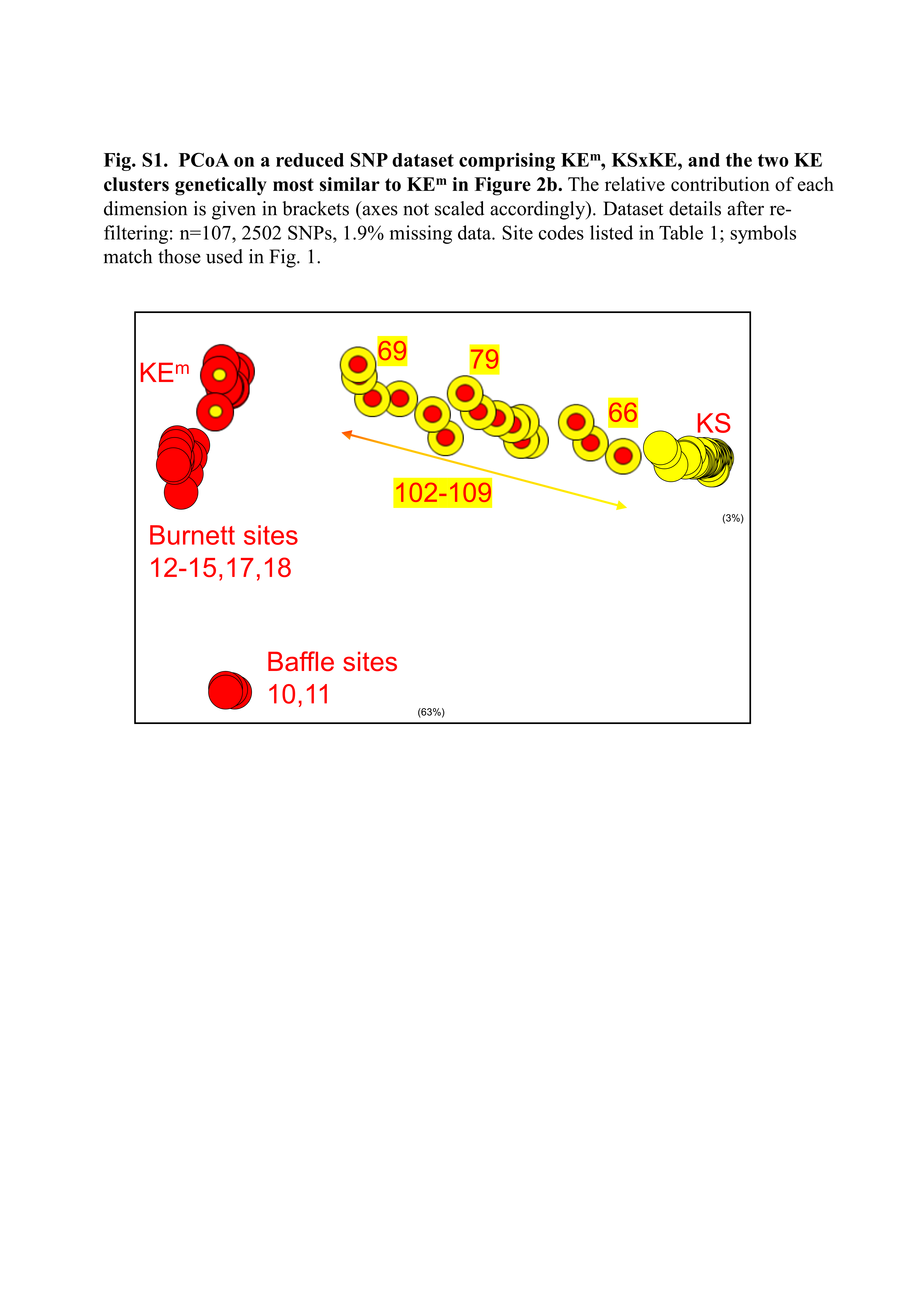

Supplement: Supplementary file 1 — Figure S1 [file ECE3-13-e10682-s005.png]

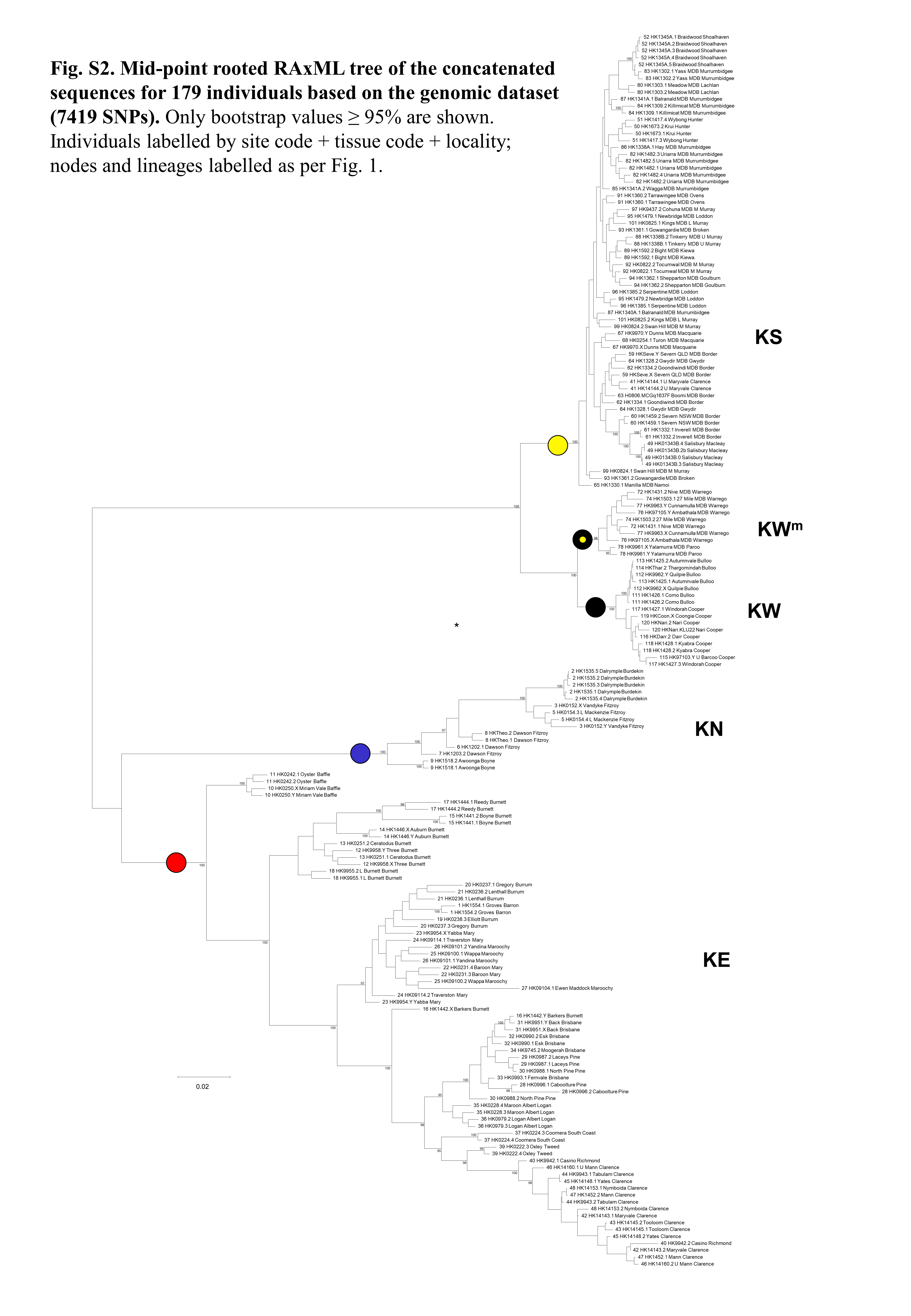

Supplement: Supplementary file 2 — Figure S2 [file ECE3-13-e10682-s003.png]

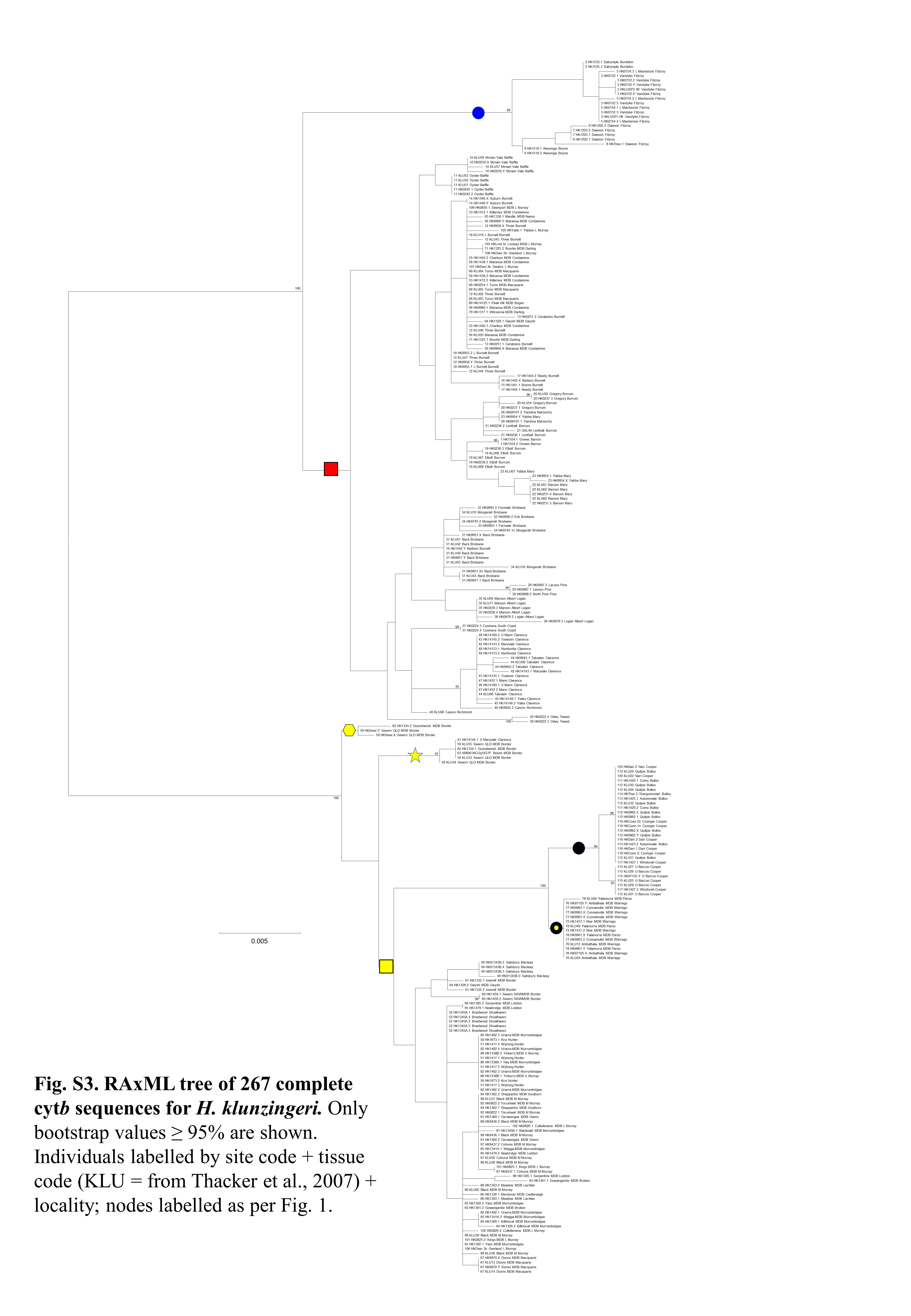

Supplement: Supplementary file 3 — Figure S3 [file ECE3-13-e10682-s004.png]

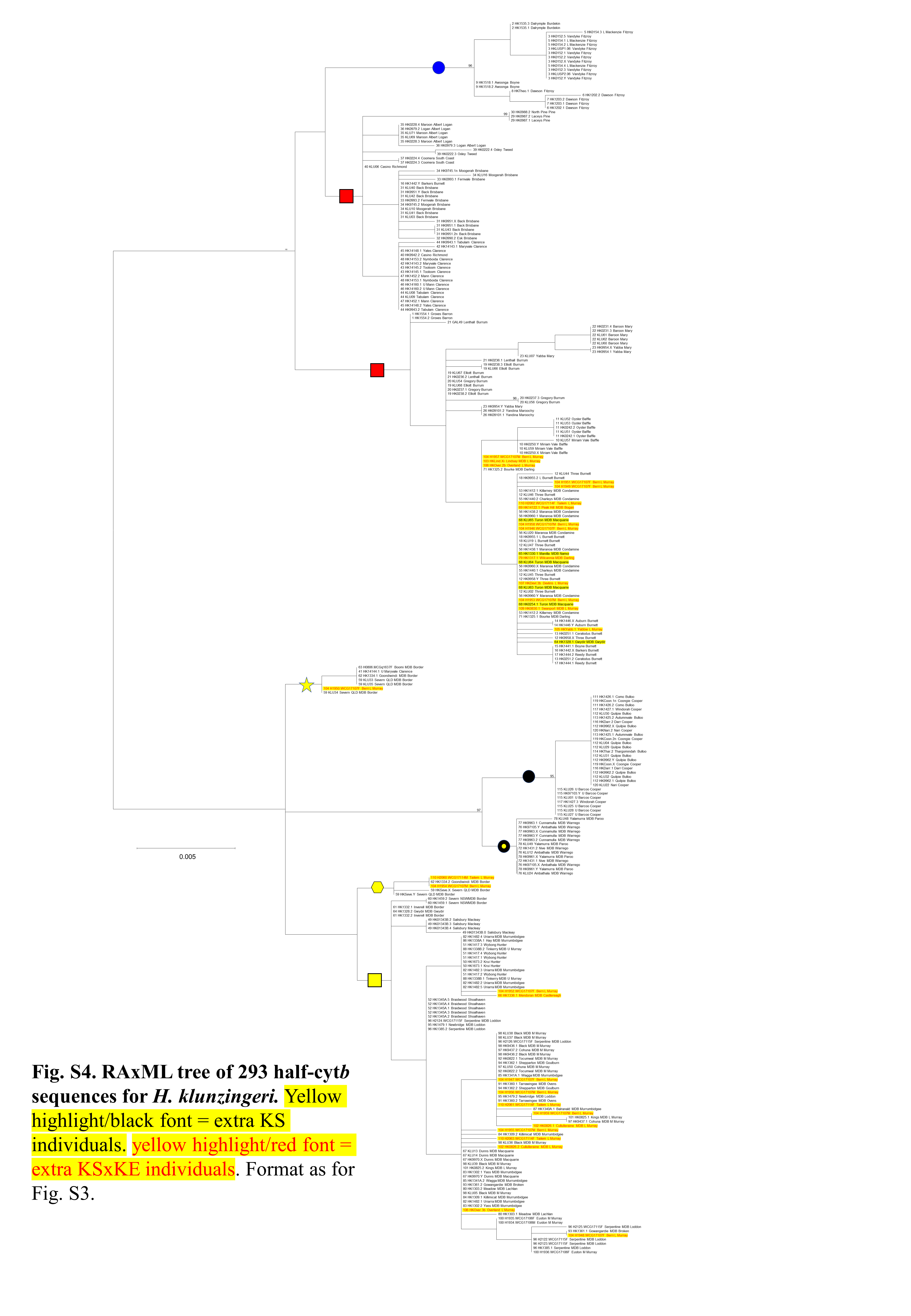

Supplement: Supplementary file 4 — Figure S4 [file ECE3-13-e10682-s001.png]

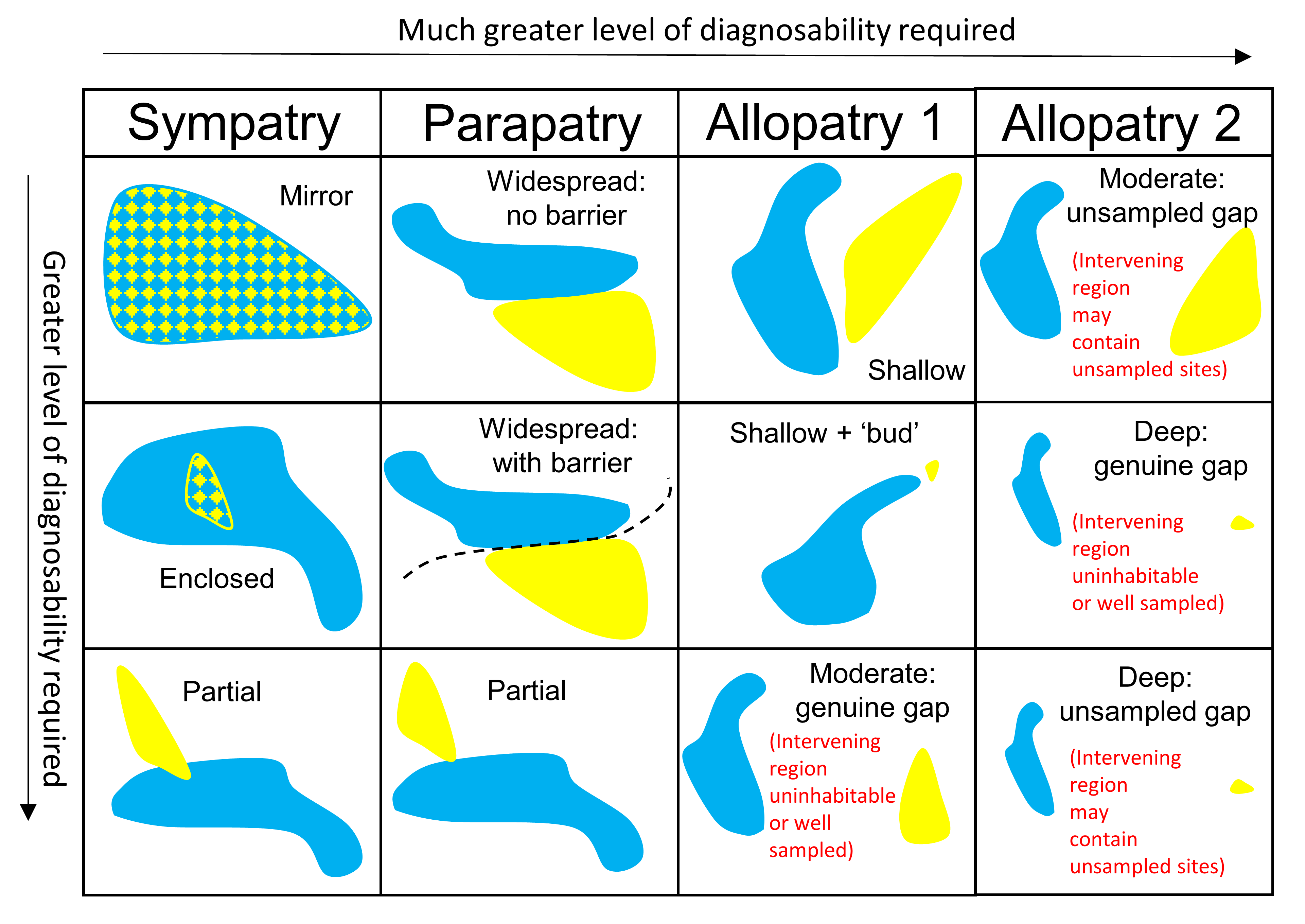

Supplement: Supplementary file 5 — Figure S5 [file ECE3-13-e10682-s006.png]
