# Supplementary material for: Phylogeography of a widespread Australian freshwater fish, western carp gudgeon (Eleotridae: Hypseleotris klunzingeri): Cryptic species, hybrid zones, and strong intra‐specific divergences
Source: Ecol Evol. 2023 Nov 1;13(11):e10682. doi: 10.1002/ece3.10682 (PMC10618717; doi:10.1002/ece3.10682)
Supplement: Supplementary file 6 — Data S1 [file ECE3-13-e10682-s002.docx]

Figure S1

PCoA on a reduced SNP dataset comprising KE^m^, KSxKE, and the two KE clusters genetically most similar to KE^m^ in Figure 2b. The relative contribution of each dimension is given in brackets (axes not scaled accordingly). Dataset details after re-filtering: n=107, 2502 SNPs, 1.9% missing data. Site codes listed in Table 1; symbols match those used in Fig. 1.

Figure S2

Mid-point rooted RAxML tree of the concatenated sequences for 179 individuals based on the genomic dataset (7419 SNPs). Only bootstrap values ≥ 95% are shown. Individuals labelled by site code + tissue code + locality; nodes and lineages labelled as per Fig. 1.

Figure S3

RAxML tree of 267 complete cyt*b* sequences for *H. klunzingeri.* Only bootstrap values ≥ 95% are shown. Individuals labelled by site code + tissue code (KLU = from Thacker et al., 2007) + locality; nodes labelled as per Fig. 1.

Figure S4

RAxML tree of 293 half-cyt*b* sequences for *H. klunzingeri.* Yellow highlight/black font = extra KS individuals. yellow highlight/red font = extra KSxKE individuals. Format as for Fig. S3.

Figure S5

Diagrammatic representation of the conceptual framework used to classify all pairwise comparisons of candidate species for their comparative geographic distributions. The terms shallow, moderate, and deep relate to how the gap distance compares to the combined geographic areas occupied by the two taxa. Modified from Unmack et al. (2022).
